# Macrocycle Unidirectional Transport Along a Linear Molecule by a Two‐Step Chemical Reaction Sequence

**DOI:** 10.1002/open.202400244

**Published:** 2024-10-29

**Authors:** Aldo C. Catalán, Lucio Peña‐Zarate, Ruy Cervantes, Alberto Vela, Jorge Tiburcio

**Affiliations:** ^1^ Department of Chemistry Center for Research and Advanced Studies (Cinvestav) Avenida IPN 2508 07360 Mexico City Mexico

**Keywords:** Molecular machines, Unidirectional motion, Metastable complex, Rotaxane, Electrostatic interactions

## Abstract

Chemical systems displaying directional motions are relevant to the operation of artificial molecular machines. Herein we present the functioning of a molecule capable of transporting a cyclic species in a preferential direction. Our system is based on a linear, non‐symmetric, positively charged molecule. This cation integrates into its structure two different reactive regions. On one side features a bulky ester group that can be exchanged by a smaller substituent; the other extreme contains an acid/base responsive moiety that plays a dual role, as part of the recognition motif and as a terminal group. In the acidic state, a dibenzo‐24‐crown‐8 ether slides into the linear component attracted by the positively charged recognition site. It does this selectively through the extreme that contains the *azepanium* group, since the other side is sterically hindered. After base addition, intermolecular interactions are lost; however, the macrocycle is unable to escape from the linear component since the energy barrier to slide over the neutral *azepane* is too large. Therefore, a metastable mechanically interlocked molecule is formed. A second reaction, now on the ester functionality, exchanges the bulky mesityl for a methyl group, small enough to allow macrocycle dissociation, completing the directional transit of the ring along the track.

## Graphical abstract

A two‐step chemical reaction sequence enables the unidirectional transport of a molecular ring along a linear molecular track. By employing an electrostatically assisted slippage approach, a stable intertwined complex is formed. This complex undergoes a rapid transformation into a metastable species through deprotonation. Transesterification of the opposite bulky group allows for unidirectional ring dissociation, with remarkable precision and control.

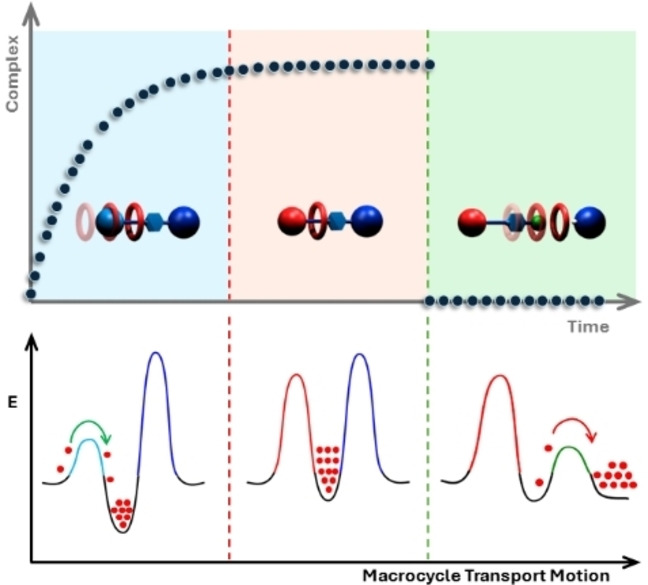

## Introduction

Directional motions are ubiquitous in nature: intracellular transport, ATP biosynthesis, bacterial motility, and muscle contraction are some biological processes exhibiting unidirectionality.^[1]^ These programmed motions are either driven by rotary or linear molecular motors. Among the latter, kinesins are a remarkable class; these protein‐based engines are responsible for transporting organelles, protein complexes and mRNAs inside the cell, by walking in a single direction through linear polymeric tracks, known as microtubules, while consuming ATP as fuel.^[2]^

For the last 30 years, chemists have been deeply interested in developing artificial analogues of these natural motors and machines, aiming to mimic its precise functioning and predictable mechanical motions.^[3]^

Amongst several artificial chemical systems, mechanically interlocked molecules, such as rotaxanes, are considered as reliable models to characterize linear motors and its operation as transporting systems.[^4^, ^5^, ^6^] Rotaxanes are chemical species formed by a linear molecule threaded by at least one macrocyclic ring; the ring is entrapped into the linear guest by mechanical bonds due to the presence of bulky end groups, larger than ring cavity, impeding its dissociation.^[6]^

In the context of rotaxanes as models for linear motors, the guest would play the role of a linear track, and the macrocyclic ring would be the cargo transported along the track, under the influence of one or several inputs. The ring would selectively enter the track through one edge, slide across and finally, abandon the track through the other extreme, describing a unidirectional trajectory relative to the linear component. So far, a handful of molecular systems have been reported, having in common the formation of a transient metastable species with rotaxane geometry, induced by chemical,^[7]^ electrical,^[8]^ or light^[9]^ stimulation.

**Figure 1:**
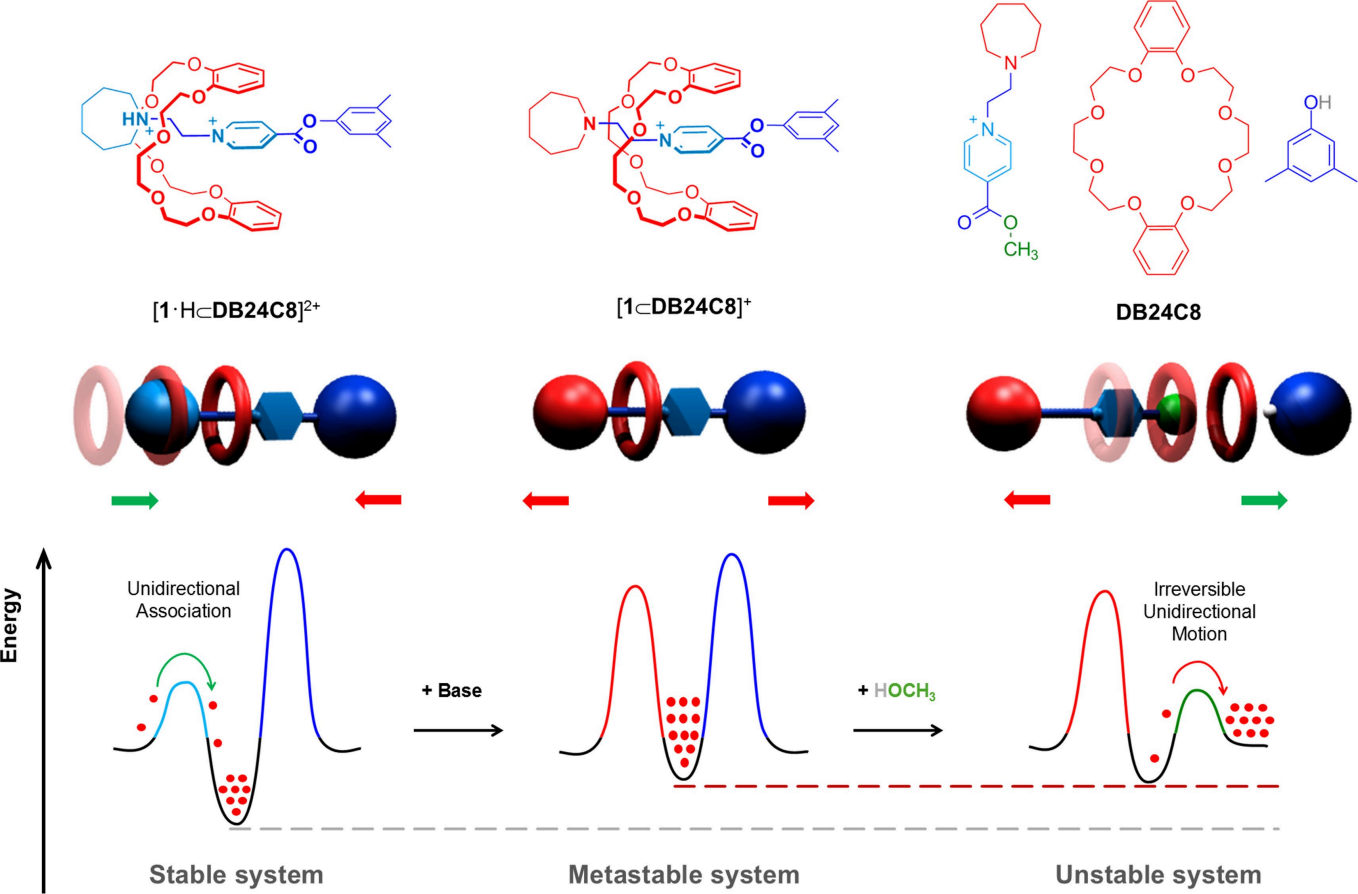
Cartoon representing the proposed unidirectional transport of a molecule (red dot) along a molecular track, alongside expected energy curve changes.

Herein we describe the design, synthesis, and performance of a linear molecule able to efficiently drive the translation of a macrocycle along its length in a preferred direction (Figure 1). We accomplished this goal by applying our reported concept: *Electrostatically Assisted Slippage Approach*,[^10^, ^11^] and two orthogonal reactions. Because of the positive charge present in the terminal seven membered *azepanium* group on the linear track, electrostatic attractive interactions with dibenzo‐24‐crown‐8 ring (**DB24C8**) are expected during the sliding process, leading to the formation of a *pseudo*‐rotaxane complex (Figure 1, left). If the proton transfer reaction is faster than complex dissociation, it must be possible to obtain a metastable [2]rotaxane species upon base addition; this interlocked system would hinder ring dissociation due to the bulkiness of the mesityl stopper and the absence of electrostatic assistance in the newly generated neutral *azepane* group (Figure 1, middle). A transesterification reaction with the appropriate alcohol would allow ring dissociation on the opposite threading side (Figure 1, right), attaining transport of the macrocycle along the guest in a single direction.

## Results and Discussion

**Scheme 1:**
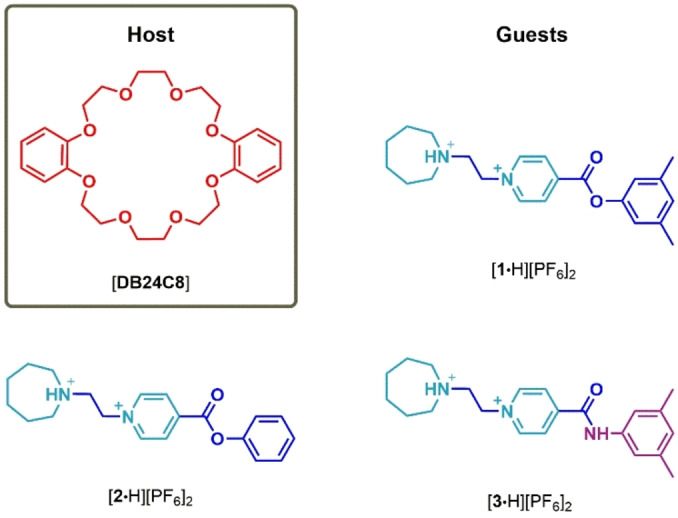
Chemical structures of macrocyclic host and linear guests.

Linear cationic guests [**1**⋅H]^2+^, [**2**⋅H]^2+^, and [**3**⋅H]^2+^ (Scheme 1) were obtained as hexafluorophosphate salts by a synthetic convergent methodology, starting from azepane, 2‐bromoethanol, *iso*‐nicotinoyl chloride, and the corresponding phenol or aniline, following our reported experimental procedure^[10]^ (see Supporting Information). All compounds were isolated as crystalline solids in good yield and were fully characterized by ^1^H and ^13^C NMR spectroscopy, and mass spectrometry.

Upon mixing guest [**1**⋅H][PF_6_]_2_ with one mole equivalent of host **DB24C8** in nitromethane‐*d_3_*, no evident changes in the ^1^H NMR spectrum are immediately observed. Only after several hours, a new set of resonances arise; no further changes in the spectrum are detected after fourteen days, indicating that chemical equilibrium has been attained (*vide infra*).

**Figure 2:**
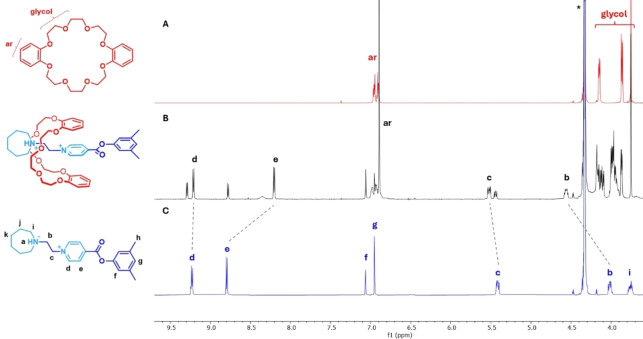
Partial stacked ^1^H NMR spectra (500 MHz, CD_3_NO_2_, 293 K, 1×10^−2^ M) of A) **DB24C8**, B) equimolar mixture of [**1**⋅H][PF_6_]_2_ and **DB24C8**, C) [**1**⋅H] [PF_6_]_2_.

The new set of resonances are indicative of the formation of an interpenetrated host−guest complex in slow exchange with the unbound species (Figure 2). A shift to high frequency for the guest ethylene protons (H_b_ +0.55 ppm and H_c_: +0.11 ppm) agrees with a series of C−H⋅⋅⋅O hydrogen bonding interactions with oxygen atoms on the crown ether, while a shift to low frequency for the *meta*‐N^+^ protons (H_e_: −0.59 ppm) suggests aromatic stacking interactions. Interestingly, the resonance for *ortho*‐N^+^ protons is only slightly disturbed (H_d_: −0.02 ppm), excluding their participation in any hydrogen bonding interactions with the host.

In addition, relative integrals in the ^1^H NMR spectrum supports the formation of a complex with a 1 : 1 stoichiometry. This observation was confirmed by experimental high‐resolution mass spectrometry; the mass spectrum shows a peak corresponding to the expected molecular ion [**1**+H+**DB24C8**]^2+^ (m/z=401.2196 amu, relative error=0.7 ppm) displaying an isotopic pattern coherent with the proposed formula.

All experimental data is consistent with a host‐guest complex adopting a *pseudo*‐rotaxane geometry [**1**⋅H⊂**DB24C8**]^2+^, with the crown ether encircling the linear guest component around the tertiary ammonium motif. A series of electrostatic, hydrogen bonding, and π‐stacking interactions are responsible for maintaining together both components. Based on integral values, 80 % of guest is involved in complex formation, providing an association constant (***K***_a_) of 1.7×10^3^ M^−1^ (Δ*G* ^o^=−18.1 kJmol^−1^), consistent with similar complexes.[^10^, ^11^]

As previously mentioned, host‐guest association proceeds slowly, over a period of fourteen days; fitting experimental data to a second order reversible reaction provides a rate constant of *k*_on_=6.3×10^−4^ M^−1^s^−1^, with an activation free energy of Δ*G*_on_^*≠*^=91.2 kJmol^−1^. Several published reports have provided strong evidence that a mesityl group functions as a stopper towards **DB24C8** threading.^[12]^ Unsurprisingly, guest [**2**⋅H]^2+^, which lacks this stopper group, immediately forms a host‐guest complex, [**2**⋅H⊂**DB24C8**]^2+^, when is combined with **DB24C8**, displaying a ***K***_a_ value of 1.9×10^3^ M^−1^ (Δ*G* ^o^=−18.4 kJmol^−1^). The new complex displays similar characteristics to [**1**⋅H⊂**DB24C8**]^2+^, but instead, chemical equilibrium is reached immediately, proving that the *azepanium* substituent on guest [**1**⋅H]^2+^ is responsible for the slow threading of **DB24C8**.

**Figure 3:**
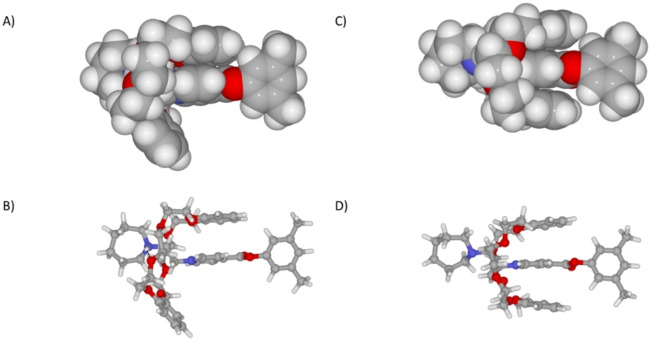
Geometry optimized structure for *pseudo*‐rotaxane complex [**1**⋅H⊂**DB24C8**]^2+^, A) van der Waals representation, B) ball‐and‐stick; and for rotaxane complex [**1**⊂**DB24C8**]^+^, C) van der Waals representation, D) ball‐and‐stick.

Computational full geometry optimization (details in the Supporting Information) offered insight into the complex structure, [**1⋅**H⊂**DB24C8**]^2+^. In the minimum energy conformation, the guest positive region *N^+^‐CH_2_‐CH_2_‐N* ^*+*^ is located inside the electron‐rich macrocycle cavity, with both ends protruding away from the ring (Figure 3, A and B). A charge‐assisted hydrogen bond between the tertiary ammonium group, on the guest, and one oxygen atom, belonging to a glycol chain on the macrocycle, is clearly observed (N−H^+^⋅⋅⋅O, 2.19 Å, 158.1°). The computed structure also displays one catechol moiety on the crown ether stacking over the pyridinium ring on the guest. Additionally, a series of weak hydrogen bonds (C−H⋅⋅⋅O) between hydrogen atoms on the guest ethylene bridge and oxygen atoms on the host cavity are detected.

Once we unambiguously established the interpenetrated nature of complex [**1⋅**H⊂**DB24C8**]^2+^, identified the non‐covalent interactions responsible of maintaining its structure and stability, and proved the selective ring threading through the *azepanium* extreme, we investigate its behavior in the presence of a base.

Addition of one equivalent of a poorly nucleophilic, but strong base, such as potassium *tert*‐butoxide (KO*t*Bu 1.0 M in *t*BuOH) to a nitromethane solution containing [**1⋅**H⊂**DB24C8**]^2+^ in chemical equilibrium with its free components, leads to a fast proton transfer reaction from the *azepanium* terminal group to the base, quantitatively converting the complex into its deprotonated form [**1**⊂**DB24C8**]^+^ (see Supporting Information). Despite the expected reduction in complex stability due to proton removal and the loss of electrostatic interactions and the N−H^+^⋅⋅⋅O hydrogen bond,^[10]^ no significant change in concentration of the complex is detected, even after fourteen days, suggesting that the singly charged complex is kinetically inert. Variations in the chemical shift of the resonances on the ^1^H NMR spectrum for the unprotonated complex [**1**⊂**DB24C8**]^+^ are indicative of a different spatial arrangement; *ortho*‐N^+^ pyridinium resonances significantly shift towards high frequency (H_d_=+0.78 ppm), whereas the *meta*‐N^+^ protons show a less pronounced shielding effect (H_e_=−0.34). These results show that deprotonation of the ammonium nitrogen in the *azepane* ring induce a rearrangement in the interpenetrated structure to maximize any remaining intermolecular interactions between host and guest.

Accordingly, full geometry optimization on the deprotonated complex [**1**⊂**DB24C8**]^+^, shows that the absence of the strong electrostatically assisted hydrogen bond drives the **DB24C8** macrocycle approximately 1 Å closer to the N atom over the pyridinium ring. This conformation maximizes C−H⋅⋅⋅O hydrogen bonds between the *ortho* hydrogen atoms on the pyridinium and the glycol oxygen atoms on the host. (Figure 3, C and D).

The kinetic inertness observed for complex [**1**⊂**DB24C8**]^+^ is due to the transformation of the *azepanium* group into a neutral *azepane* substituent. Stoddart^[13]^ and Loeb^[14]^ have previously shown that the energy barrier to slip **DB24C8** over a neutral seven‐member ring is too large to overcome at room temperature. This also holds true for a neutral nitrogen‐containing *azepane* ring, with similar dimensions.[^10^, ^11^] At this point, the **DB24C8** ring is kinetically trapped in the linear component, even when a significant reduction in stability is expected for a singly charged guest, as observed for complex [**2**⊂**DB24C8**]^+^, with a ***K***_a_=2.8×10^1^ M^−1^ and a Δ*G* ^o^=−8.1 kJmol^−1^ (see Supporting Information).

However, addition of a non‐hindered alcohol, such as methanol, to a solution containing the complex [**1**⊂**DB24C8**]^+^ triggers the immediate substitution of the mesityl terminal group by a methyl group, allowing fast and complete ring dethreading, giving rise to unbound macrocycle **DB24C8**, free singly‐charge guest and 3,5‐dimethylphenol. Further proof of this process was obtained by utilizing guest [**3⋅**H]^2+^ featuring an amide bond between the pyridinium and the phenyl substituent. In the presence of host **DB24C8**, this guest forms a *pseudo*‐rotaxane complex [**3⋅**H⊂**DB24C8**]^2+^ in solution, with similar thermodynamic and kinetic characteristics to complex [**1⋅**H⊂**DB24C8**]^2+^ (see Supporting Information). Due to the robustness of the amide bond in complex [**3**⊂**DB24C8**]^2+^, base and methanol addition does not provoke any changes, allowing the complex to indefinitely persist in solution.

**Figure 4:**
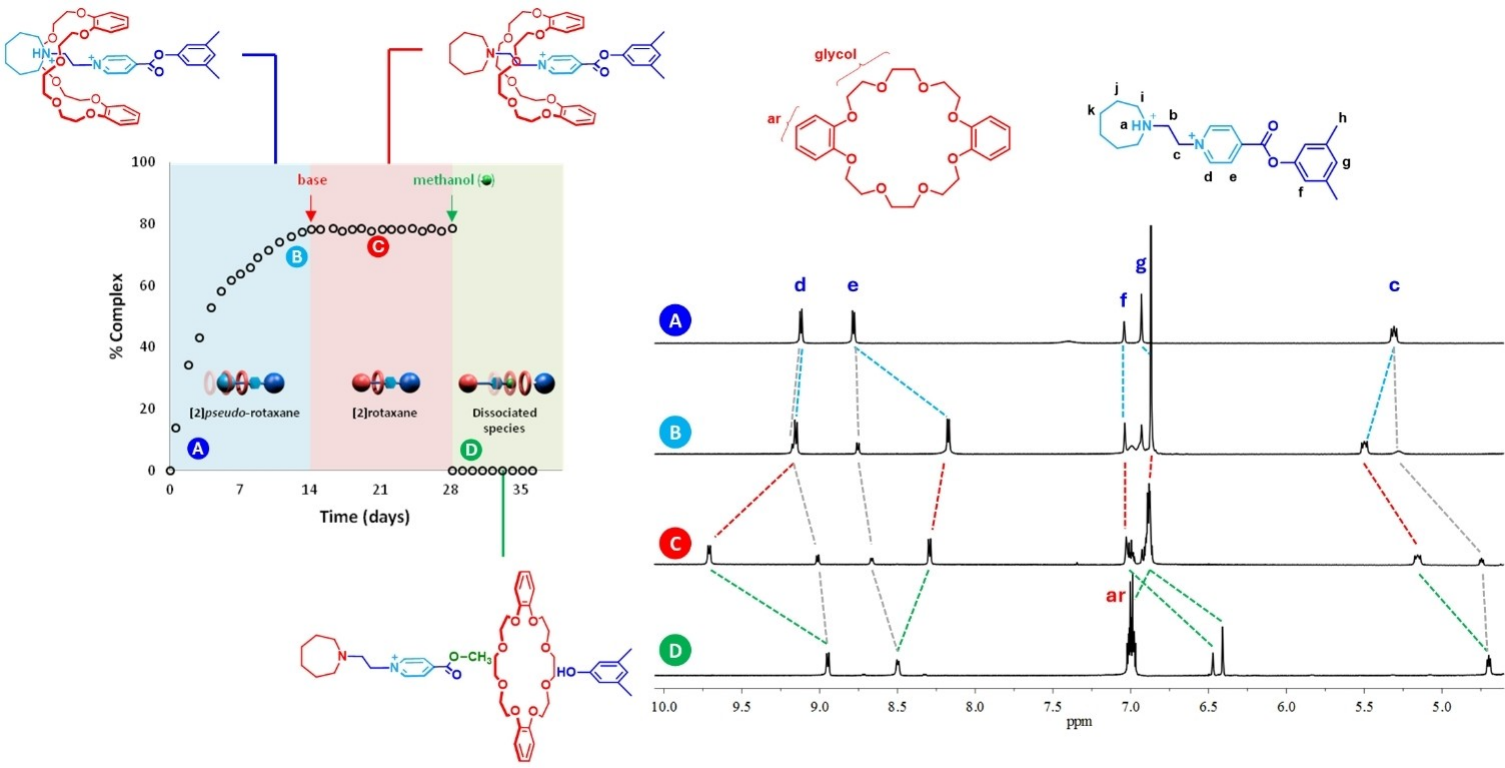
Partial ^1^H NMR spectra (500 MHz, CD_3_NO_2_, 293 K, [**host**]=[**guest**]=1×10^−2^ M). (**A**) [**1**⋅H][PF_6_]_2_. (**B**) An equimolar mixture of [**1**⋅H][PF_6_]_2_ and **DB24C8** at equilibrium. (**C**) The previous described system after addition of one equivalent of potassium *tert*‐butoxide. (**D**) The same system after addition of 100 equivalents of methanol. Counterions were omitted. Lines relate changes in the resonances.

Figure 4 summarizes the full unidirectional macrocycle transport along the linear guest driven by a set of two chemical reactions. We initiate with a solution containing equimolar concentrations of unbound macrocycle and guest (A); over a period of fourteen days, about 80 % of the rings are selectively threaded by the guest through the *azepanium* group, affording a [2]*pseudo*‐rotaxane complex in solution (B). After base addition, a fast proton transfer reaction occurs, from the *azepanium* moiety located on the guest to the base, giving rise to a neutral *azepane* group; this group impedes ring dissociation, generating a metastable [2]rotaxane species, capable to persist over a long period of time (C). By adding methanol to this solution, a transesterification reaction takes place, swapping the bulky mesityl group by a methyl substituent; since this group is rather small, dissociation occurs over this guest terminus releasing the macrocycle back into the solution, thus completing the unidirectional transit of the macrocycle over the guest (D).

The same two‐step chemical reaction sequence, *i. e*., deprotonation/transesterification, was applied to guest [**1**⋅H]^2+^ and the result is a linear molecule where the methyl group is joined to the ester function and 3,5‐dimethylphenol. Although in principle, a transesterification reaction can be reversed by the addition of the corresponding alcohol in the presence of base,^[15]^ in our case it was impossible to regenerate the starting guest component [**1⋅**H]^2+^ from [**1**]^+^. Addition of another equivalent of base to the system induces the formation of a nitroalkane anion by reacting with the solvent. Consecutively, the formation of a carbon‐carbon bond between the produced nitroalkane and the hydrolyzed linear component followed a nitro aldolic reaction, better known as Henry reaction.^[16]^ Regardless of the amount of phenol added to re‐attach the stopper at the end of the linear component, it is impossible to regenerate the initial component (see Supporting Information).

## Conclusions

We have demonstrated that is possible to direct the transit of a macrocyclic ring along a linear asymmetric molecular track by means of two consecutive chemical reactions. The linear component integrates an *azepanium* terminal group to slide the crown ether ring, assisted by electrostatic attractive effects; slipping across the opposite terminal group is avoided since a mesityl substituent is bulky enough to prevent crown ether sliding. Once the ring is embedded into the linear fragment, electrostatic interactions are canceled by means of a proton transfer reaction with a base, generating a mechanically interlocked metastable complex. A second reaction (transesterification) replaces the mesityl stopper by a smaller methyl group, driving ring selective directional dissociation. Unfortunately, this system cannot be reversed; development of reversible systems utilizing a variation of this approach is currently in progress in our group.

## Conflict of Interests

The authors declare no conflict of interest.

1

## Supporting information

As a service to our authors and readers, this journal provides supporting information supplied by the authors. Such materials are peer reviewed and may be re‐organized for online delivery, but are not copy‐edited or typeset. Technical support issues arising from supporting information (other than missing files) should be addressed to the authors.

Supporting Information

## Data Availability

The data that support the findings of this study are available in the supplementary material of this article.
