# Supplementary material for: Macrocycle Unidirectional Transport Along a Linear Molecule by a Two‐Step Chemical Reaction Sequence
Source: ChemistryOpen. 2024 Oct 29;14(1):e202400244. doi: 10.1002/open.202400244 (PMC12128140; doi:10.1002/open.202400244)
Supplement: Supplementary file 1 — Supporting Information [file OPEN-14-e202400244-s001.pdf]

ChemistryOpen

Supporting Information

Macrocycle Unidirectional Transport Along a Linear Molecule by a Two-Step Chemical Reaction Sequence

Aldo C. Catalán,* Lucio Peña-Zarate, Ruy Cervantes, Alberto Vela, and Jorge Tiburcio*

Supporting Information

Macrocycle Unidirectional Transport Along a Linear Molecule by a Two-step Chemical Reaction Sequence

Aldo C. Catalán,* Lucio Peña-Zarate, Ruy Cervantes, Alberto Vela, and Jorge Tiburcio*

1. **General information** 2

2. **Synthesis and characterization of guest compounds** 2

   2.1 Compound [**1**·H][PF_6]$_2$ 3

   2.2 Compound [**2**·H][PF_6]$_2$ 9

   2.3 Compound [**3**·H][PF_6]$_2$ 14

3. **[2]*Pseudo*-rotaxanes** 19

   3.1 [**1**·H⊂**DB24C8**]$^{2+}$ 19

   3.2 [**2**·H⊂**DB24C8**]$^{2+}$ 23

   3.3 [**3**·H⊂**DB24C8**]$^{2+}$ 25

4. **Computational analysis** 28

5. **Transesterification reaction** 31

6. **References** 33

1. General information

NMR spectra were recorded on either JEOL ECA 500 MHz or Bruker AVANCE III 400 MHz spectrometers locked to the deuterated solvent. Mass spectra were obtained on an Agilent G1969A electrospray ionization mass time-of-flight (ToF) spectrometer. A PolyScience thermostat circulation bath was used for temperature control for the rate constant experiments. Curve fitting for rate constants determinations was determined with OriginPro 2020.

Chemical reagents were obtained from Sigma-Aldrich and used as received. All reactions were carried out in an open atmosphere and reagent grade solvents were used.

2. Synthesis and characterization of guest compounds

Synthesis of precursor 1-(2-bromoethyl)azepanium bromide was achieved by modifying a previously reported procedure [S1-S2] with 40% isolated product yield.

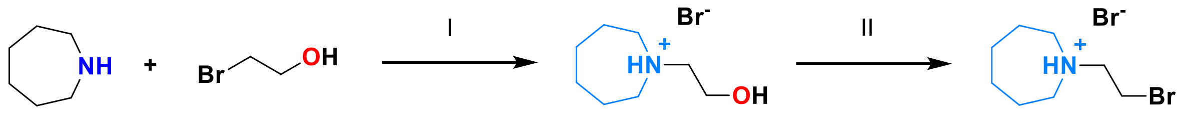

Scheme S1. Synthesis of 1-(2-bromoethyl)azepanium bromide.

I) Azepane, (1 mL, 8.8 mmol) was dissolved in 5 mL of acetonitrile, then 2-bromoethanol (1.5 mL, 20.1 mmol) was added dropwise. The solution was heated at reflux temperature for 36 hours. After reaching room temperature (aprox. 20°C), diethyl ether was added and a white crystalline solid precipitate. The solid was filtered off and washed repeatedly with cold acetone to yield 1.54 g of a white crystalline solid in a 78% yield.

II) 1-(2-hidroxietil)azepano (0. 5 g, 2.2 mmol) was dissolved in 3 mL of HBr 48% and heated at reflux temperature, coupled to a Dean-Stark apparatus, for 24 h. After cooling to room temperature, acetone is added to precipitate a brownish solid. The solid was filtered off and washed with acetone to yield a white solid (0.54 g 85%).

2.1 Compound [**1**·H][PF_6]$_2$

3,5-dimethylphenyl isonicotinate was synthesized following a previously described method. [S3]

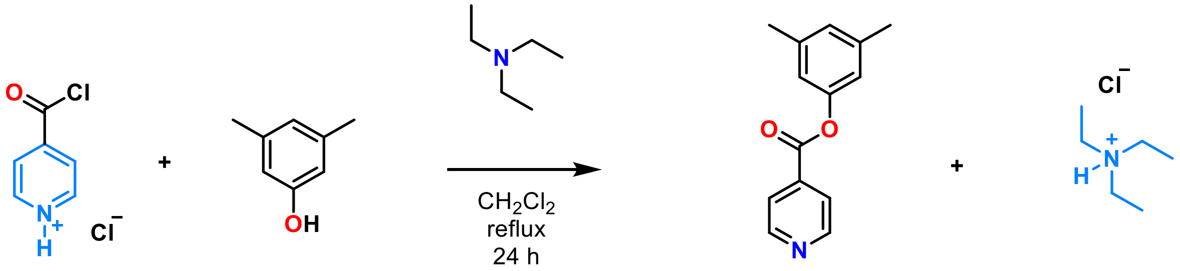

Scheme S2. Synthesis of 3,5-dimethylphenyl isonicotinate.

To a solution containing 0.67 g of 3,5-dimethylphenol (5.5 mmol) in CH_2Cl_2 (8 mL) was added 2.13 g of isonicotinoyl chloride hydrochloride (12.1 mmol). Just after mixing, 5 mL of triethylamine (48 mmol) dissolved in CH_2Cl_2 (8 mL) was added drop wise while stirring. The mixture was heated at reflux temperature for 24 hours. The solid was separated by filtration and the solution was washed repeatedly with water (6 × 15 mL). After drying the dichloromethane solution with Na_2SO_4 anhydrous and rota evaporation a yellowish oil was obtained which solidified as a white solid (1.12g, 90%). The 3,5-dimethylpheny isonicotinate was utilized for the synthesis of compound [**1**·H][PF_6]$_2$ without further purification.

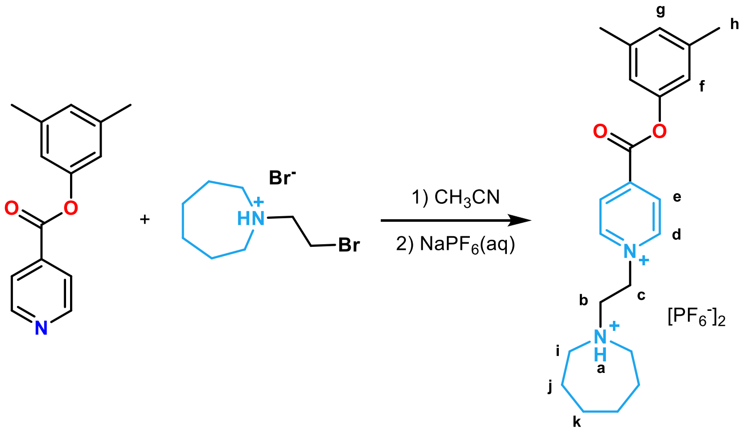

Scheme S3. Synthesis of **[1·H][PF$_6$]$_2$**.

1-(2-bromoethyl)azepanium bromide, 0.26 g (0.9 mmol), and 3,5-dimethylphenyl isonicotinate, 1.08 g (4.8 mmol) were heated at reflux temperature in acetonitrile (5 mL) for 72 hours. The pale-yellow solid was filtered off and washed with chloroform (0.30g, 64%). The bromide salt can be transformed into the hexafluorophosphate salt by anion exchange in the presence of 1 equivalent of HBr (0.27g, 72%).

ESI-TOF HRMS: *m*/*z* found for [**1**+H]$^{2+}$ 177.1156, calculated 177.1148, error 4.5 ppm.

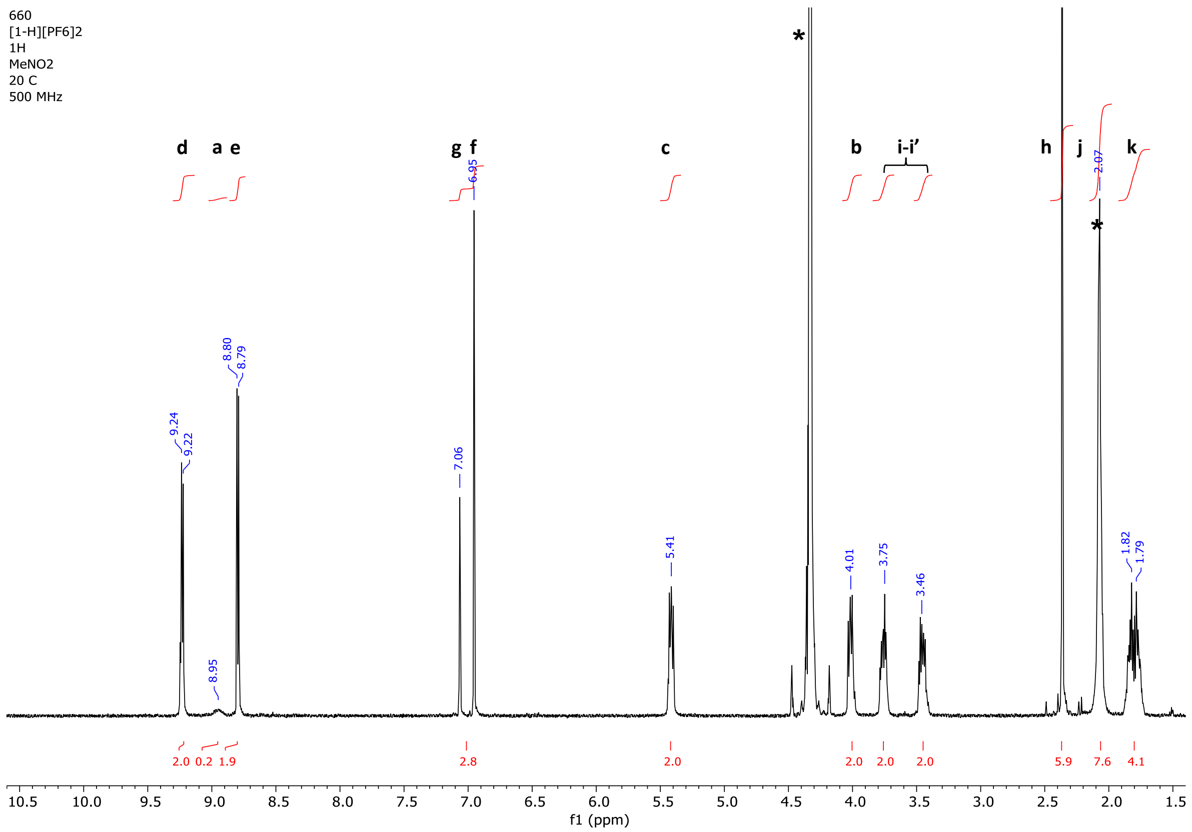

Figure S1. Assigned ^1H NMR spectrum of the guest [**1**·H][PF_6]$_2$ (500 MHz, CD_3NO_2, * = residual solvents).

^1H NMR (500 MHz, CD_3NO_2) δ_H 9.23 (2H, d, $^3J_{d\text{-}e}$ = 7.0 Hz, H_d), 8.95 (1H, s, H_a), 8.80 (2H, d, $^3J_{e\text{-}d}$ = 7.0 Hz, H_e), 7.06 (1H, m, H_g), 6.95 (2H, m, H_f), 5.41 (2H, m, H_c), 4.01 (2H, m, H_b), 3.75 (2H, m, H_i), 3.46 (2H, m, $H_{i'}$), 2.36 (6H, m, H_h), 1.95-2.15 (4H, m, H_j), 1.7-1.9 (4H, m, H_k).

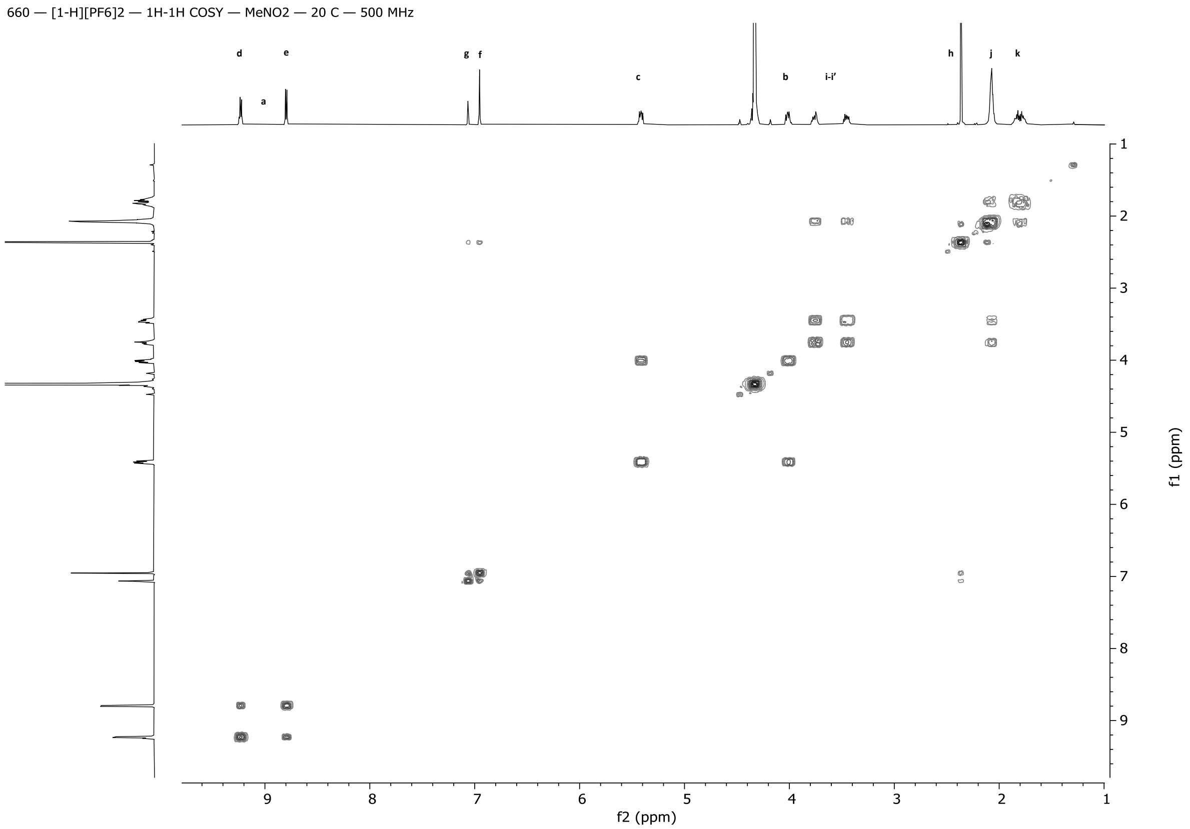

Figure S2. ^{1}H-^{1}H COSY NMR spectrum of the guest [**1**·H][PF_6]$_2$ (500 MHz, CD_3NO_2).

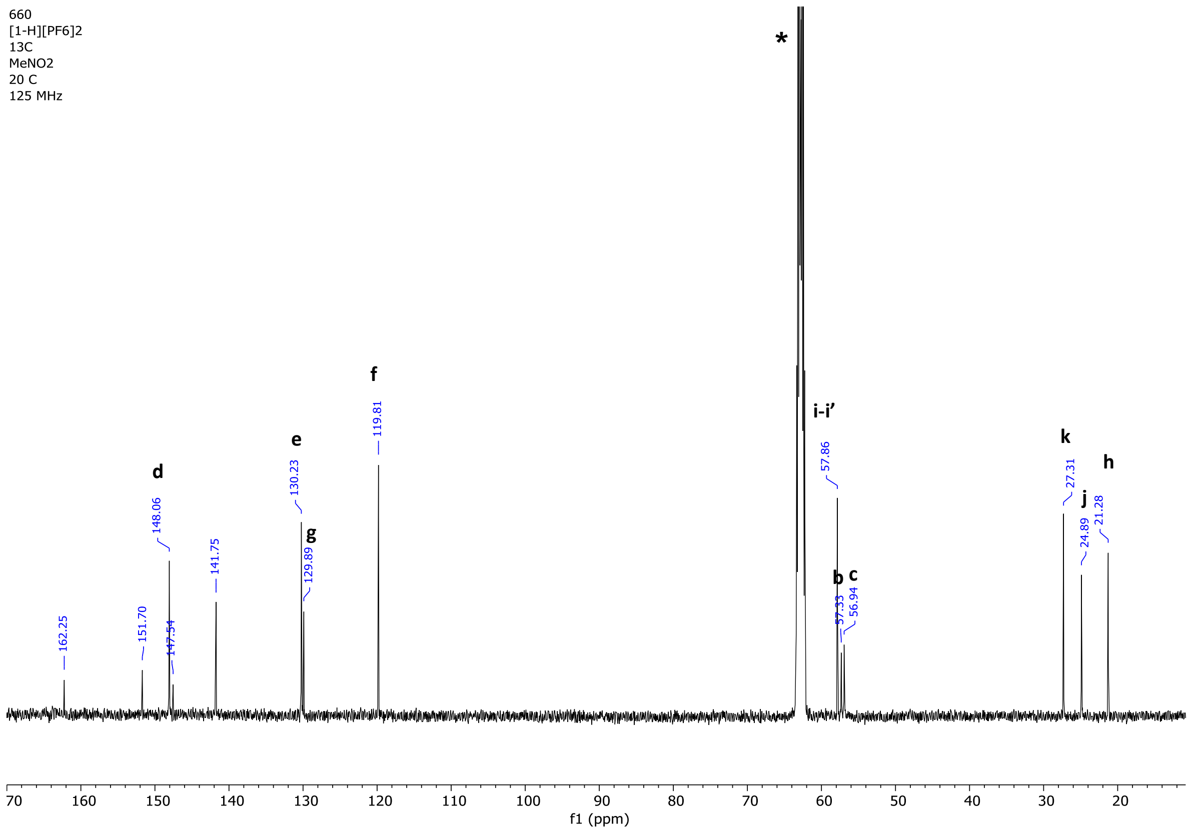

Figure S3. Assigned ^{13}C NMR spectrum of the guest [**1**·H][PF_6]$_2$ (125 MHz, CD_3NO_2, * = residual solvents).

^{13}C NMR (125 MHz, CD_3NO_2) δ_C 162.25 ($C_{carbonyl}$); 151.70 (C_{ipso}); 148.06 (C_d); 147.54 (C_{ipso}); 141.75 (C_{ipso}); 130.23 (C_e); 129.89 (C_g); 119.81 (C_f); 57.86 (C_i); 57.33 (C_b); 56.94 (C_c); 27.31 (C_k); 24.89 (C_j); 21.28 (C_h).

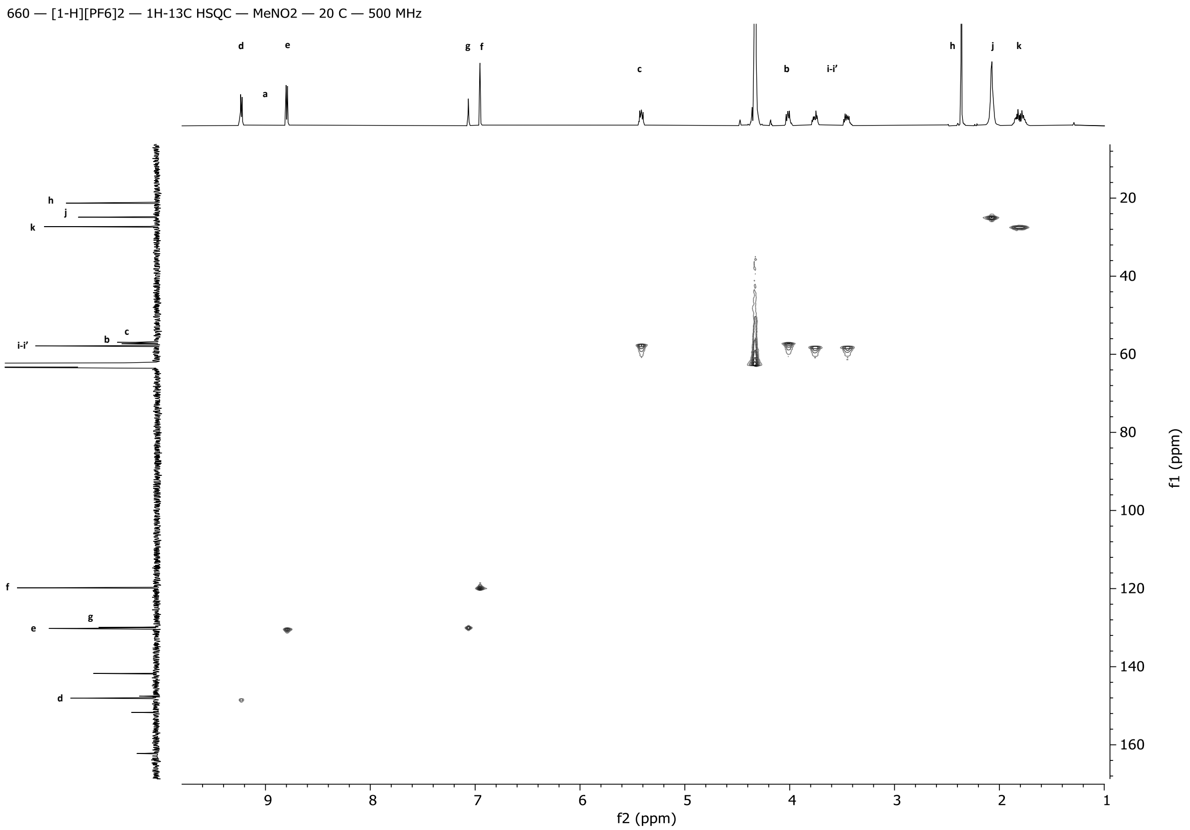

Figure S4. ^{1}H-^{13}C HSQC NMR spectrum of the guest [**1**·H][PF_6]$_2$ (400-125 MHz, CD_3NO_2).

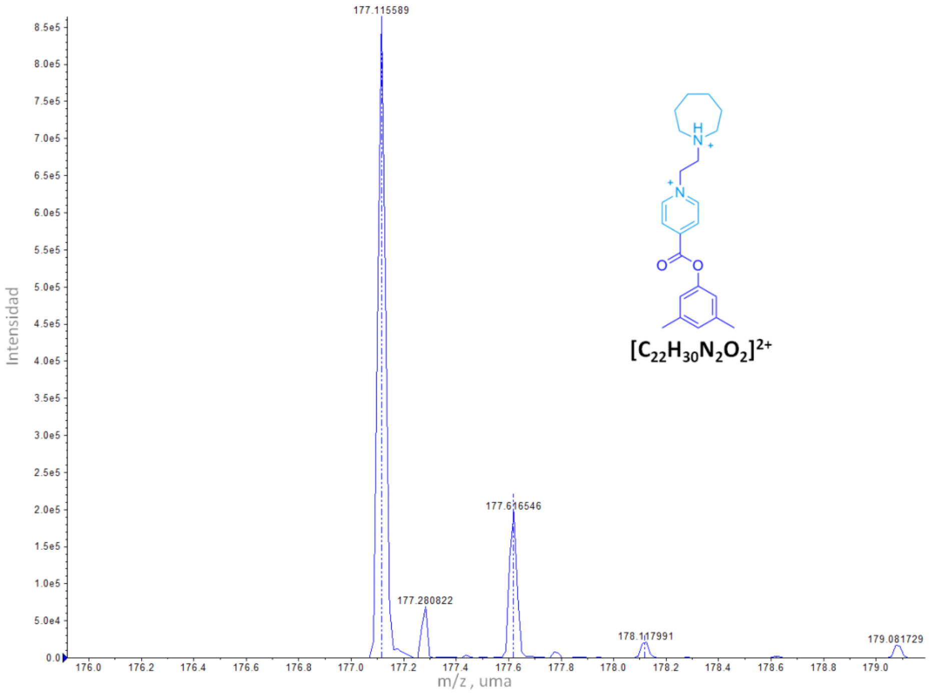

Figure S5. ESI-TOF mass histogram of guest [**1**·H][PF_6]$_2$. Experimental, solid line; calculated, dashed line.

2.2 Compound [**2**·H][PF_6]$_2$

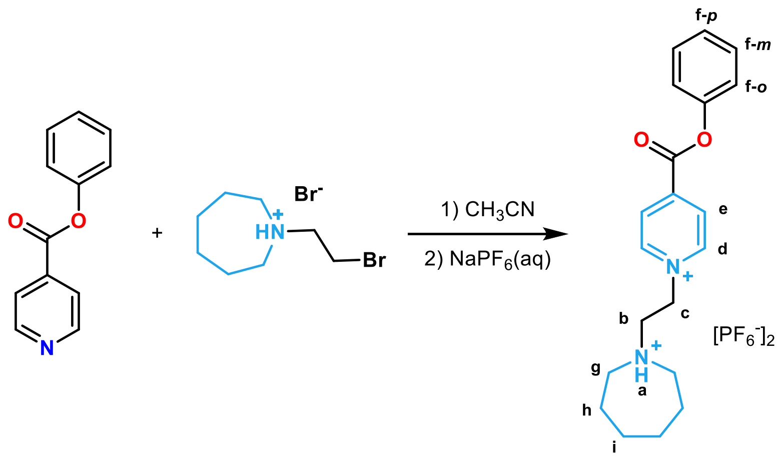

Scheme S4. Synthesis of [**2**·H][PF_6]$_2$.

1-(2-bromoethyl)azepanium bromide, 0.26 g (0.9 mmol), and phenyl isonicotinate, 0.96 g (4.8 mmol) were heated at reflux temperature in acetonitrile (5 mL) for 72 hours. The pale-yellow solid was filtered off and washed with chloroform (0.30 g, 70%). The bromide salt can be transformed into the hexafluorophosphate salt by anion exchange with $NaPF_6$ in the presence of 1 equivalent of HPF_6 (0.31 g, 80%).

ESI-TOF MS: *m/z* found for [**2**]$^+$ 325.2, calculated 325.2.

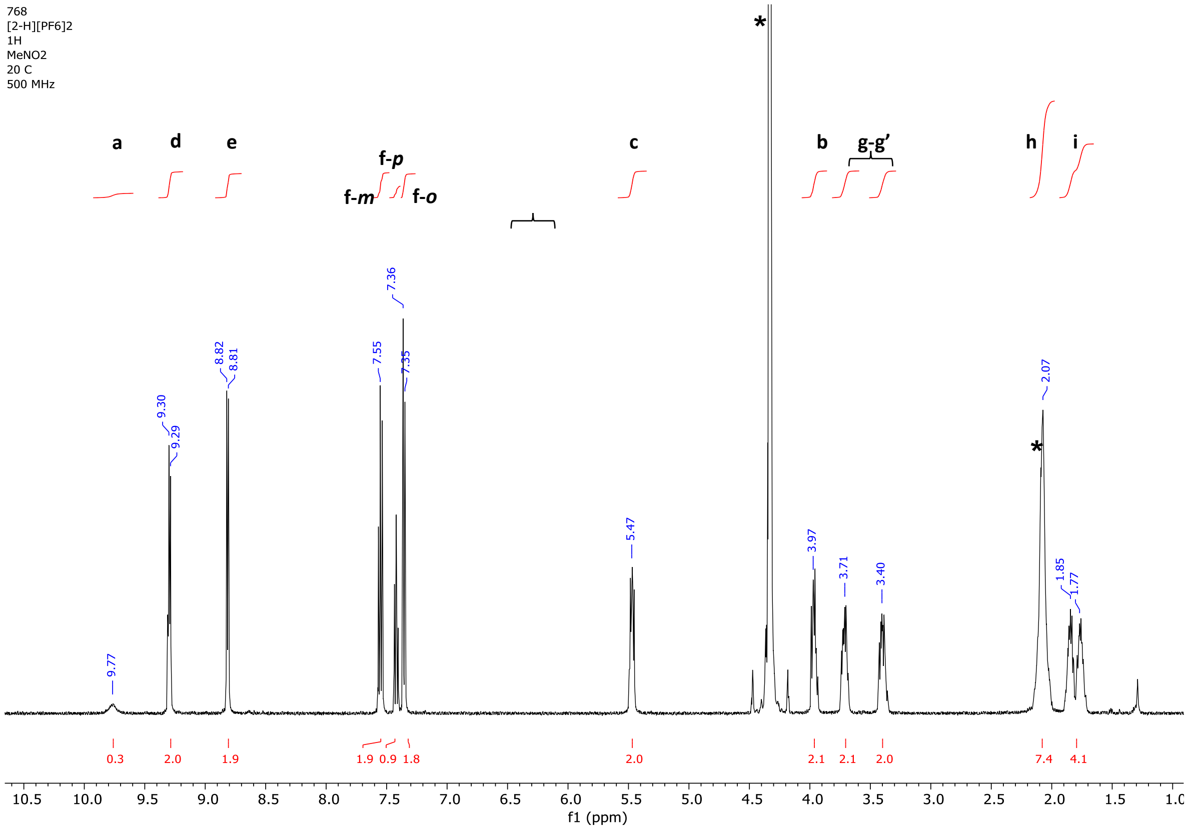

Figure S6. Assigned ^{1}H NMR spectrum of the guest [**2**·H][PF_6]$_2$ (500 MHz, CD_3NO_2, * = residual solvents).

^{1}H NMR (500 MHz, CD_3NO_2) δ_H 9.77 (1H, s, H_a), 9.29 (2H, d, $^3J_{d\text{-}e}$ = 6.5 Hz, H_d), 8.81 (2H, d, $^3J_{e\text{-}d}$ = 6.5 Hz, H_e), 7.55 (2H, m, H_f-*m*), 7.42 (1H, m, H_f-*p*), 7.35 (2H, m, H_f-*o*), 5.47 (2H, m, H_c), 3.97 (2H, m, H_b), 3.71 (2H, m, H_g), 3.40 (2H, m, $H_{g'}$) 2.2-1.9 (4H, m, H_h), 1.9-1.7 (4H, m, H_i).

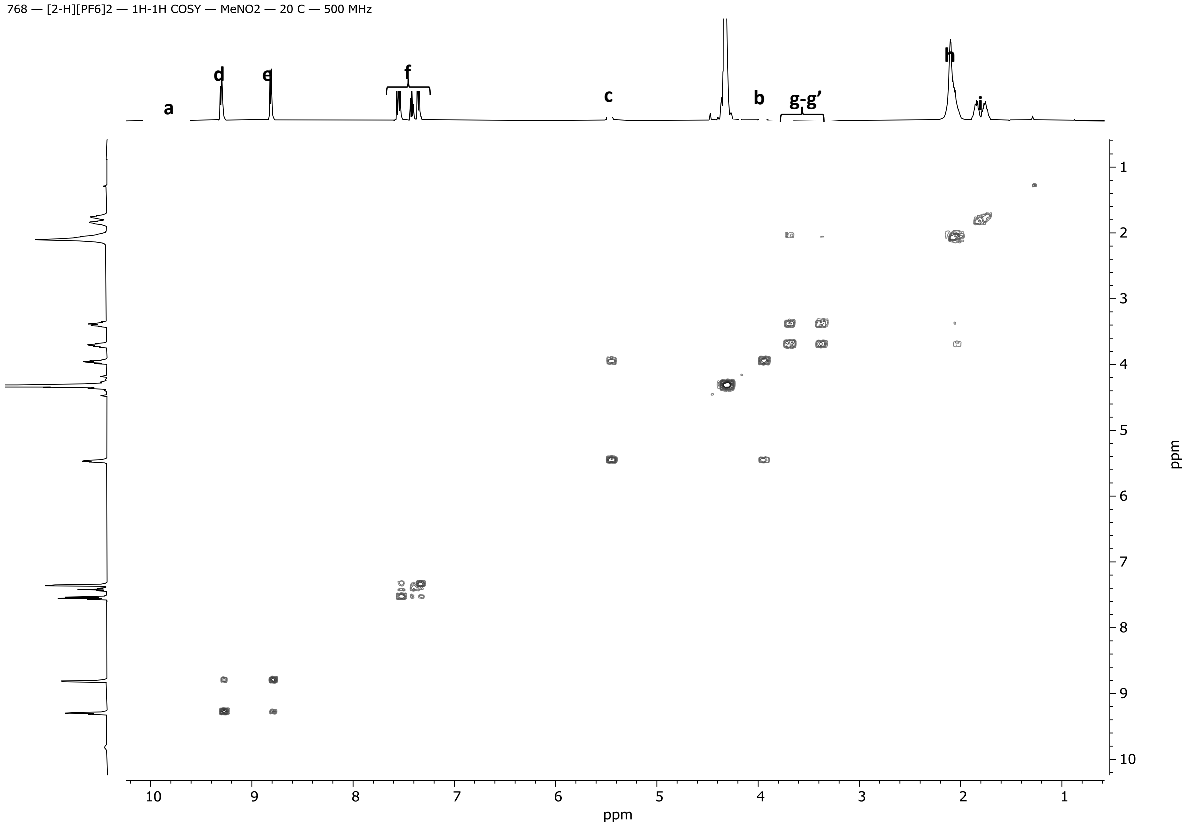

Figure S7. ^{1}H-^{1}H COSY NMR spectrum of the guest [**2**·H][PF_6]$_2$ (500 MHz, CD_3NO_2).

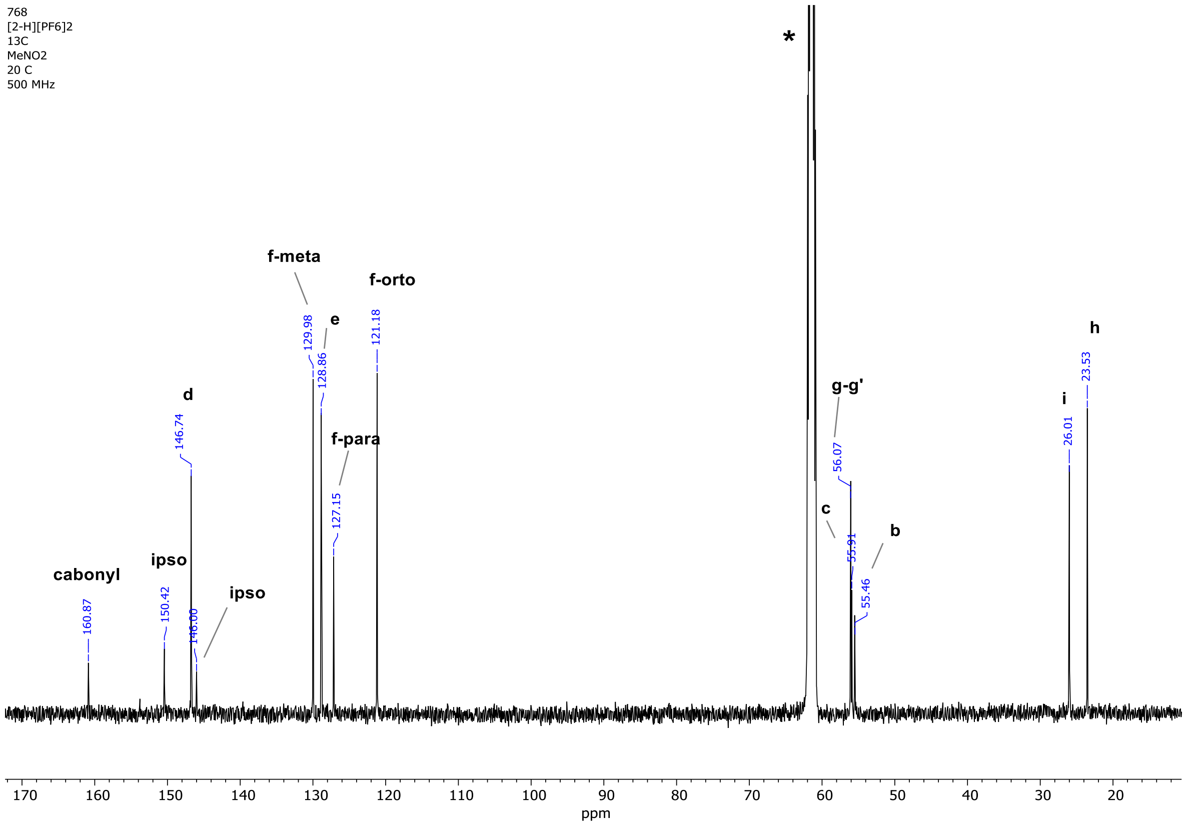

Figure S8. Assigned ^{13}C NMR spectrum of the guest [**2**·H][PF_6]$_2$ (125 MHz, CD_3NO_2, * = residual solvents).

^{13}C NMR (125 MHz, CD_3NO_2) δ_C 160.87 ($C_{carbonyl}$); 150.42 (C_{ipso}); 146.74 (C_d); 146.00 (C_{ipso}); 129.98 ($C_{f\text{-}meta}$); 128.86 (C_e); 127.15 ($C_{f\text{-}para}$); 121.18 ($C_{f\text{-}ortho}$); 55.91 (C_c); 55.49 (C_b); 56.07 ($C_{g\text{-}g'}$); 26.01 (C_i); 23.53 (C_h).

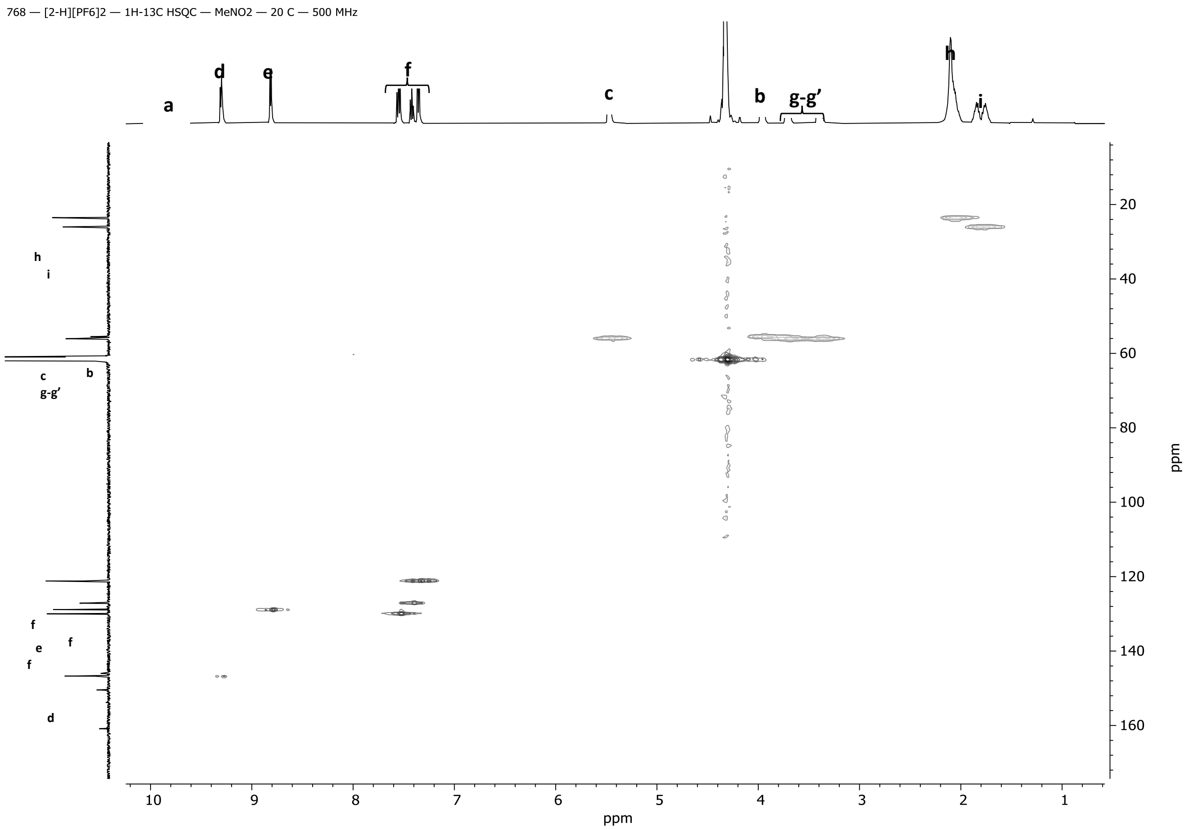

Figure S9. ^{1}H-^{13}C HSQC NMR spectrum of the guest [**2**·H][PF_6]$_2$ (400-125 MHz, CD_3NO_2).

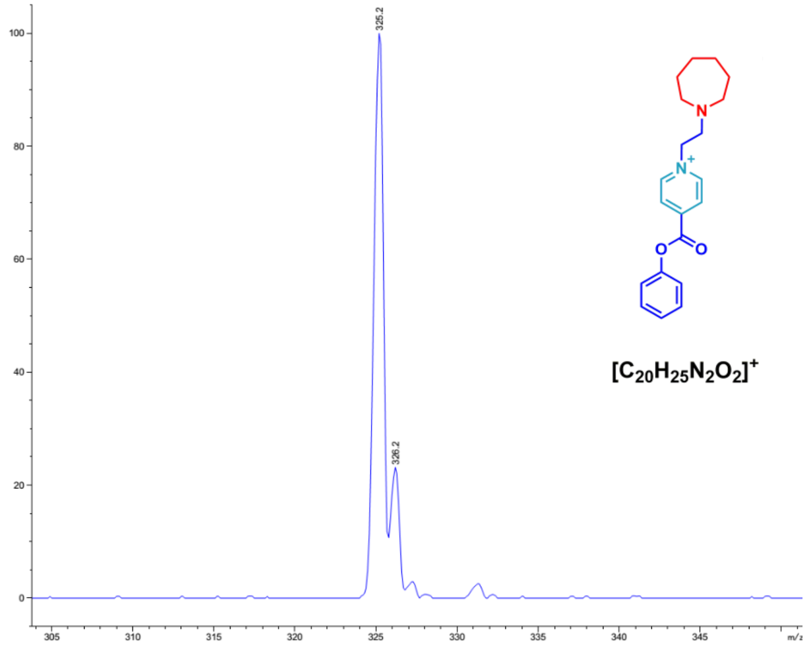

Figure S10. ESI-TOF mass histogram of guest [**2**·H][PF_6]$_2$.

2.3 Compound [**3**·H][PF_6]$_2$

The dumbbell precursor *N*-(3,5-dimethylphenyl)isonicotinamide was synthesised modifying a previously reported procedure.[S4]

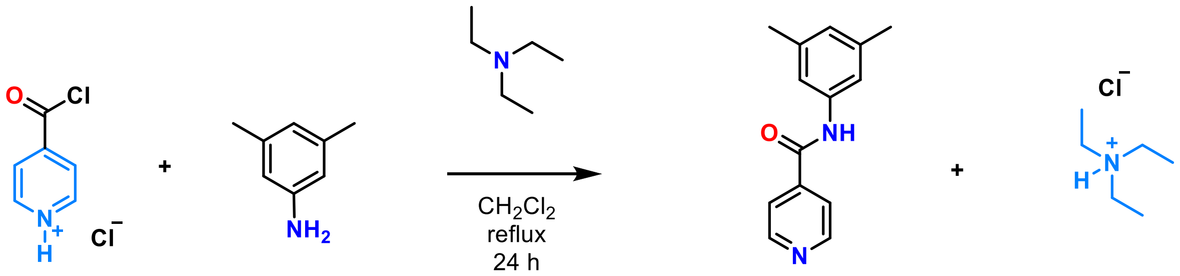

Scheme S5. Synthesis of the *N*-(3,5-dimethylphenyl)isonicotinamide.

To a solution containing 0.6 mL of 3,5-dimethylaniline (4.8 mmol) in dichloromethane (8.0 mL) was added 1.0 g of isonicotinoyl chloride hydrochloride (5.3 mmol). Just after mixing, 3.0 mL of triethylamine (21.3 mmol) dissolved in dichloromethane (8.0 mL) was added drop wise. The mixture was refluxed for one day. The solid was separated by filtration and the filtrate was washed repeatedly with water. After the organic layer had dried (Na_2SO_4 anhydrous), the solvent was evaporated yielding a yellowish solid (0.8 g, 70 %). The *N*-(3,5-dimethylphenyl)isonicotinamide was utilized for the next synthesis without further purification.

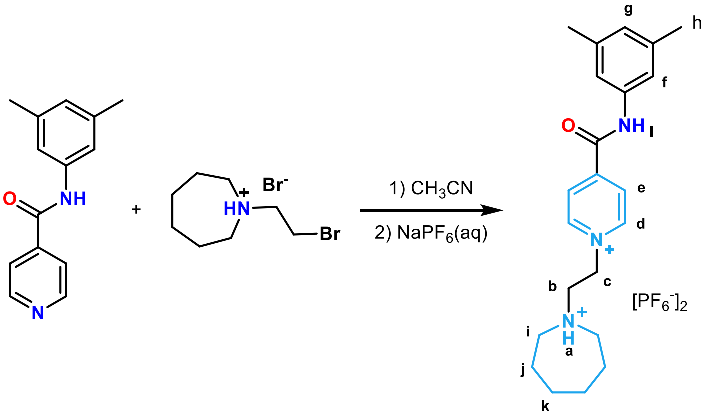

Scheme S6. Synthesis of the guest [**3**·H][PF_6]$_2$.

1-(2-bromoethyl)azepanium bromide compound (0.3 g, 0.9 mmol) was mixed with *N*-(3,5-dimethylphenyl)isonicotinamide (0.6 g, 2.7 mmol) in acetonitrile (12.0 mL). The mixture was refluxed for seven days. The bright yellow solid was filtered and washed with ethanol (0.2 g, 41%). The bromide salt was transformed into the hexafluorophosphate salt by anion exchange in the presence of 1 equivalent of hydrobromic acid (0.2 g, 80 %).

ESI-TOF HRMS: *m/z* found for $[\mathbf{3}+H]^{2+}$ 176.6238, calculated 176.6228.

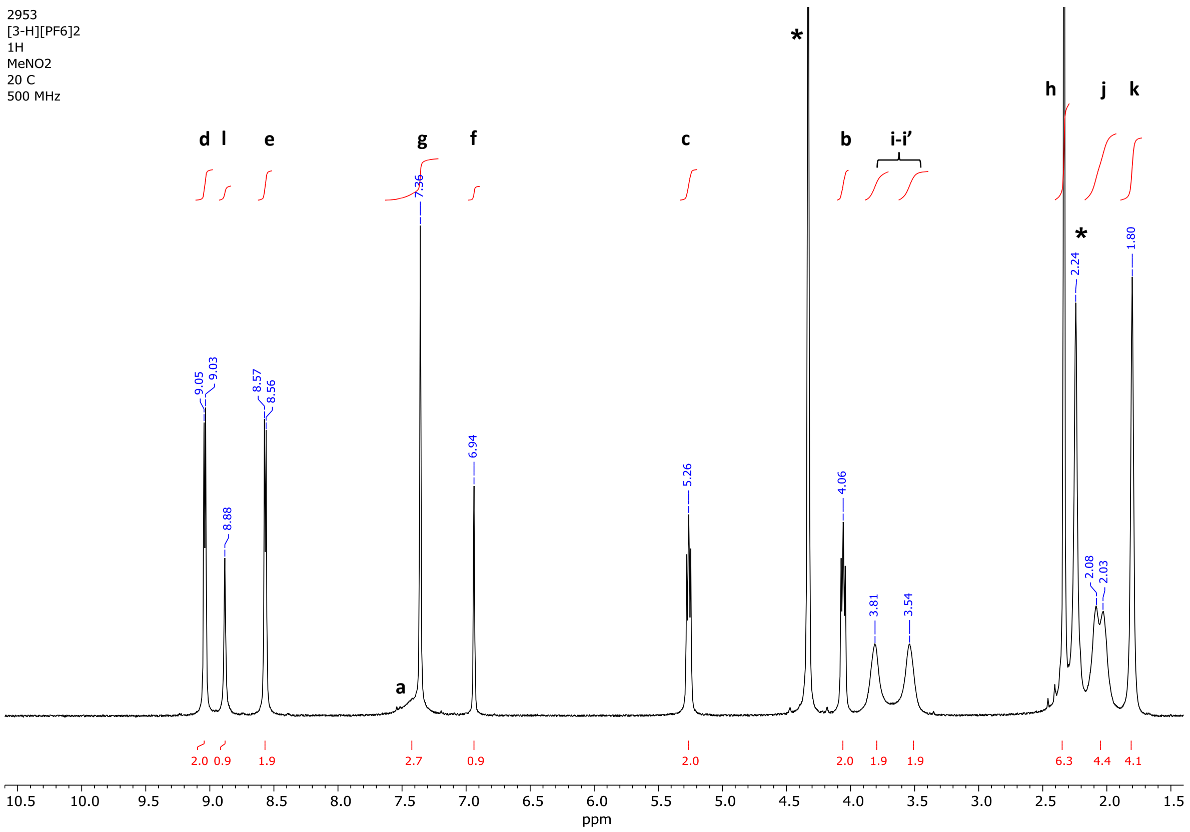

Figure S11. Assigned ^1H NMR spectrum of the guest $[\mathbf{3}\cdot H][PF_6]_2$ (500 MHz, CD_3NO_2, * = residual solvents).

^1H NMR (500 MHz, CD_3NO_2) δ_H 9.04 (2H, d, $^3J_{d\text{-}e}$ = 6.0 Hz, H_d), 8.88 (1H, s, H_l), 8.57 (2H, d, $^3J_{e\text{-}d}$ = 6.0 Hz, H_e), 7.43 (1H, br.s, H_a), 7.36 (2H, s, H_f), 6.94 (1H, s, H_g), 5.26 (2H, m, H_c), 4.06 (2H, m, H_b), 3.81-3.53 (4H, br.m, H_i), 2.33 (6H, s, H_h), 2.06 (4H, br.m, H_j), 1.80 (4H, br.s, H_k).

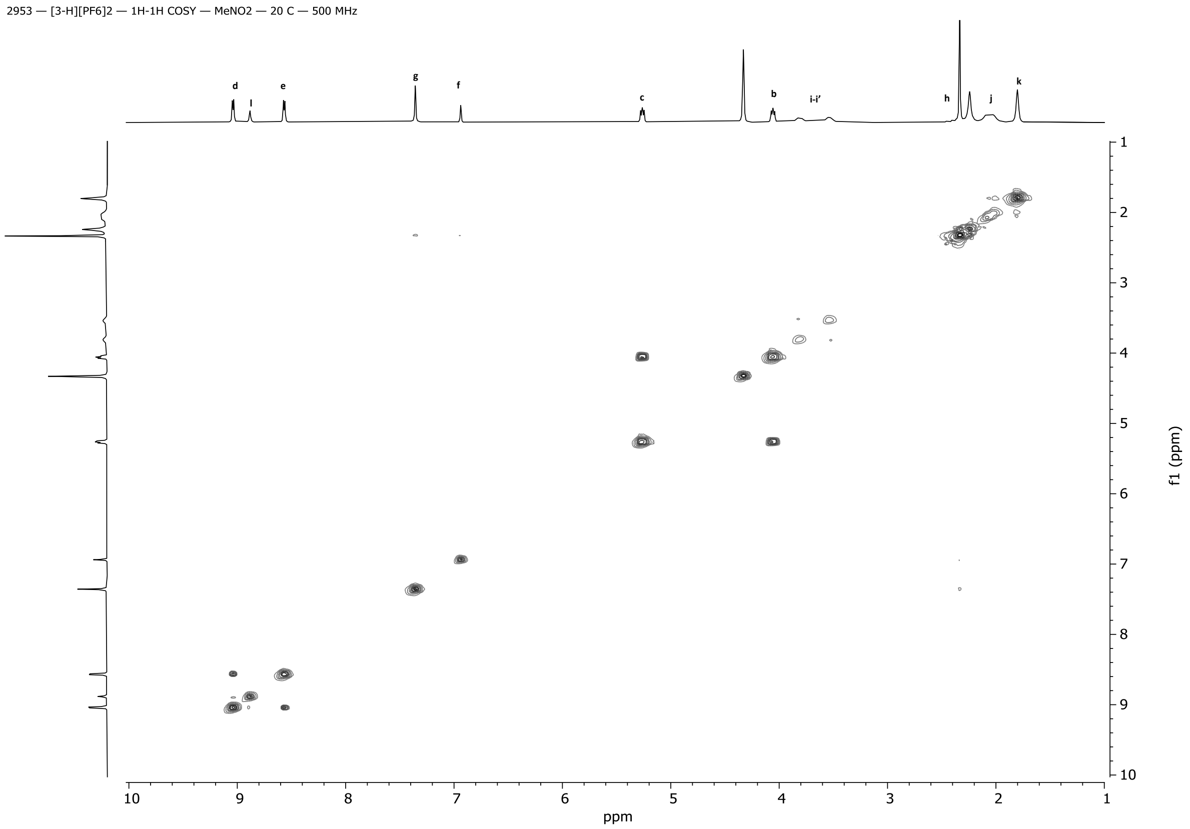

Figure S12. ^{1}H-^{1}H COSY NMR spectrum of the guest [**3**·H][PF_6]$_2$ (500 MHz, CD_3NO_2).

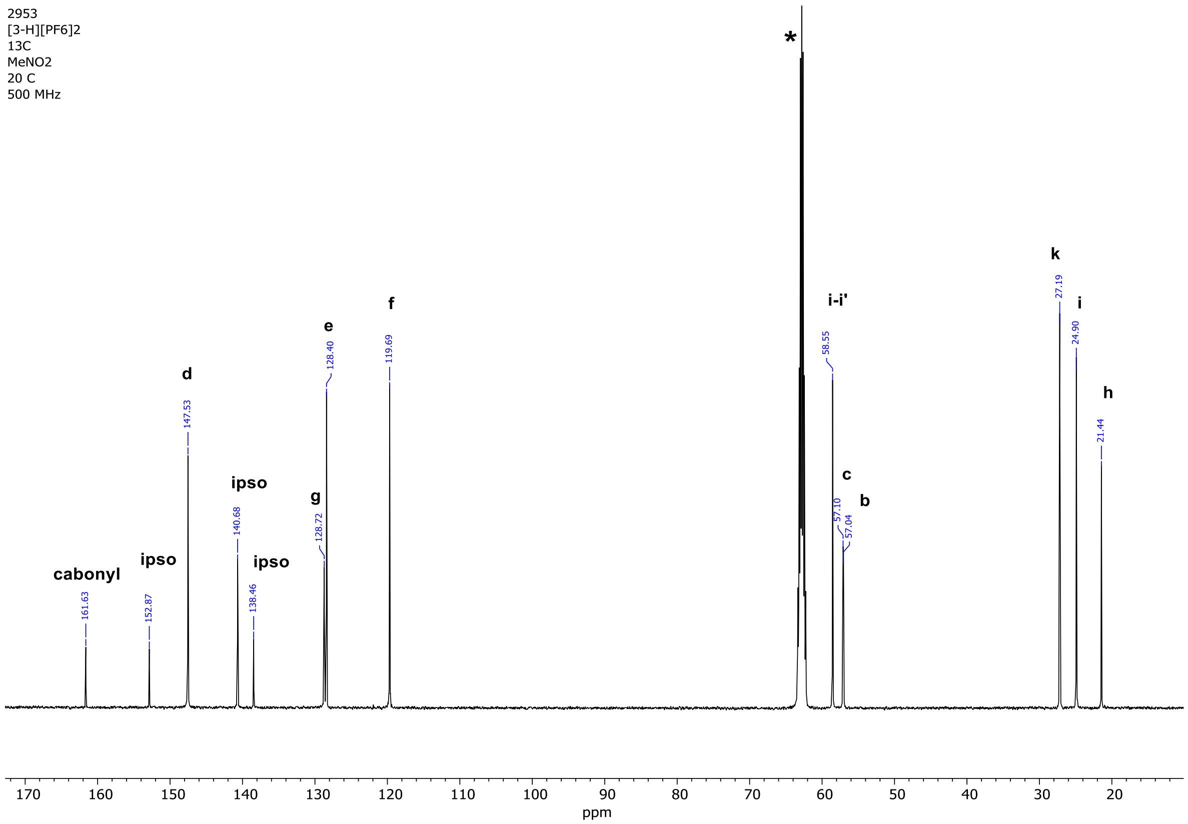

Figure S13. Assigned ^{13}C NMR spectrum of the guest [**3**·H][PF_6]$_2$ (125 MHz, CD_3NO_2, * = residual solvents).

^{13}C NMR (125 MHz, CD_3NO_2) δ_C 161.63 ($C_{carbonyl}$); 152.87 (C_{ipso}); 147.53 (C_d); 140.68 (C_{ipso}); 138.46 (C_{ipso}); 128.72 (C_g); 128.40 (C_e); 119.69 (C_f); 58.55 (Ci-i'); 57.10 (C_c); 57.04 (C_b); 27.19 (C_k); 24.90 (C_j); 21.44 (C_h). ESI-HRMS: *m*/*z* found for [**3**+H]$^{2+}$ 176.6268, calculated 176.6228, error 5.7 ppm.

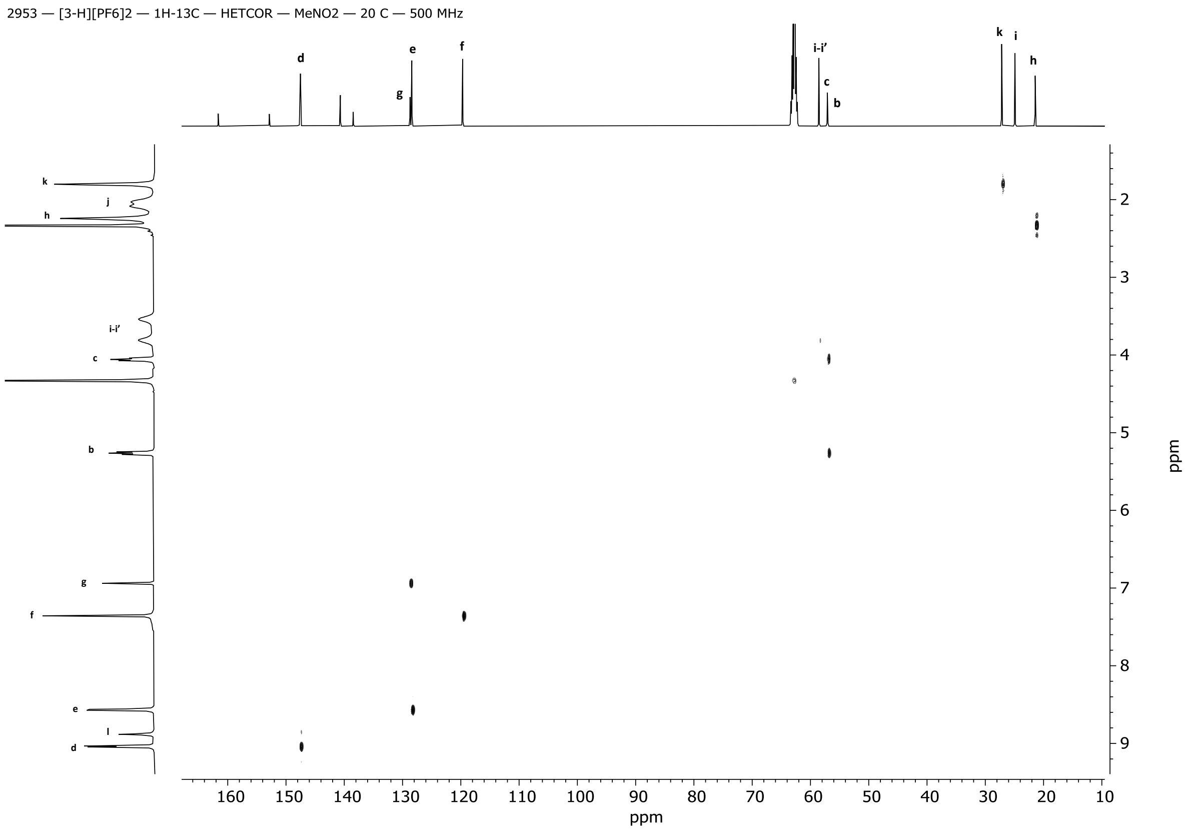

Figure S14. ^{1}H-^{13}C HETCOR NMR spectrum of the guest [**3**·H][PF_6]$_2$ (400-125 MHz, CD_3NO_2).

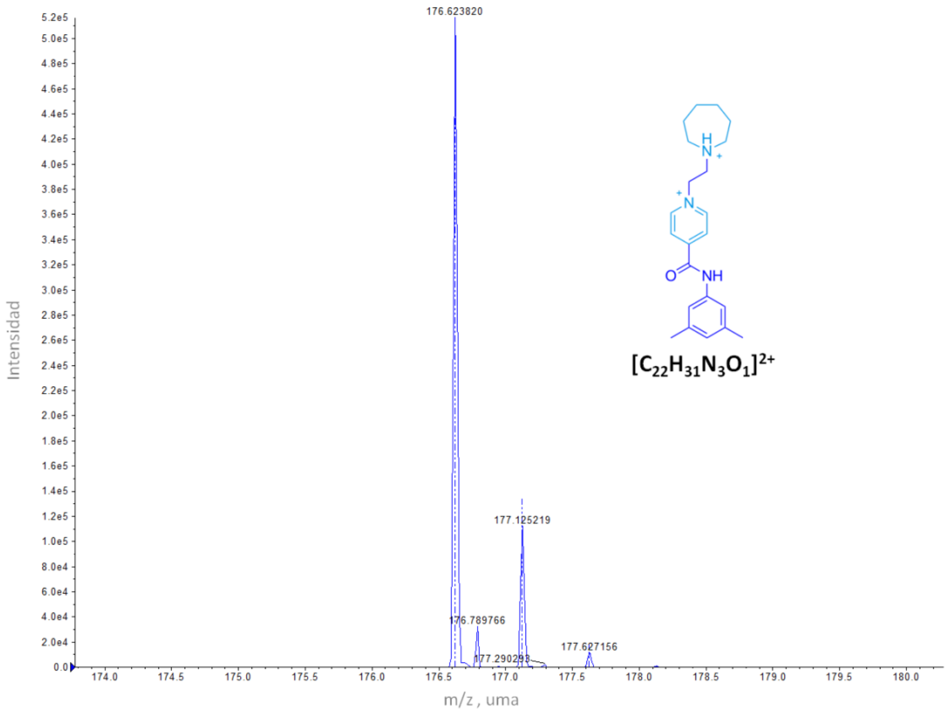

Figure S15. ESI-TOF mass histogram of guest [**3**·H][PF_6]$_2$. Experimental, solid line; calculated, dashed line.

3. [2]*Pseudo*-rotaxanes

3.1 [**1**·H⊂**DB24C8**]$^{2+}$

Upon mixing two equimolar solutions of guest [**1**·H][PF_6]$_2$ and host **DB24C8** in CD_3NO_2 a pale-yellow solution was observed. It took 14 days to reach equilibrium. At this time, the ^{1}H NMR spectrum showed the formation of an interpenetrated compound in chemical equilibrium with its non-complexed species (Figure S16 d).

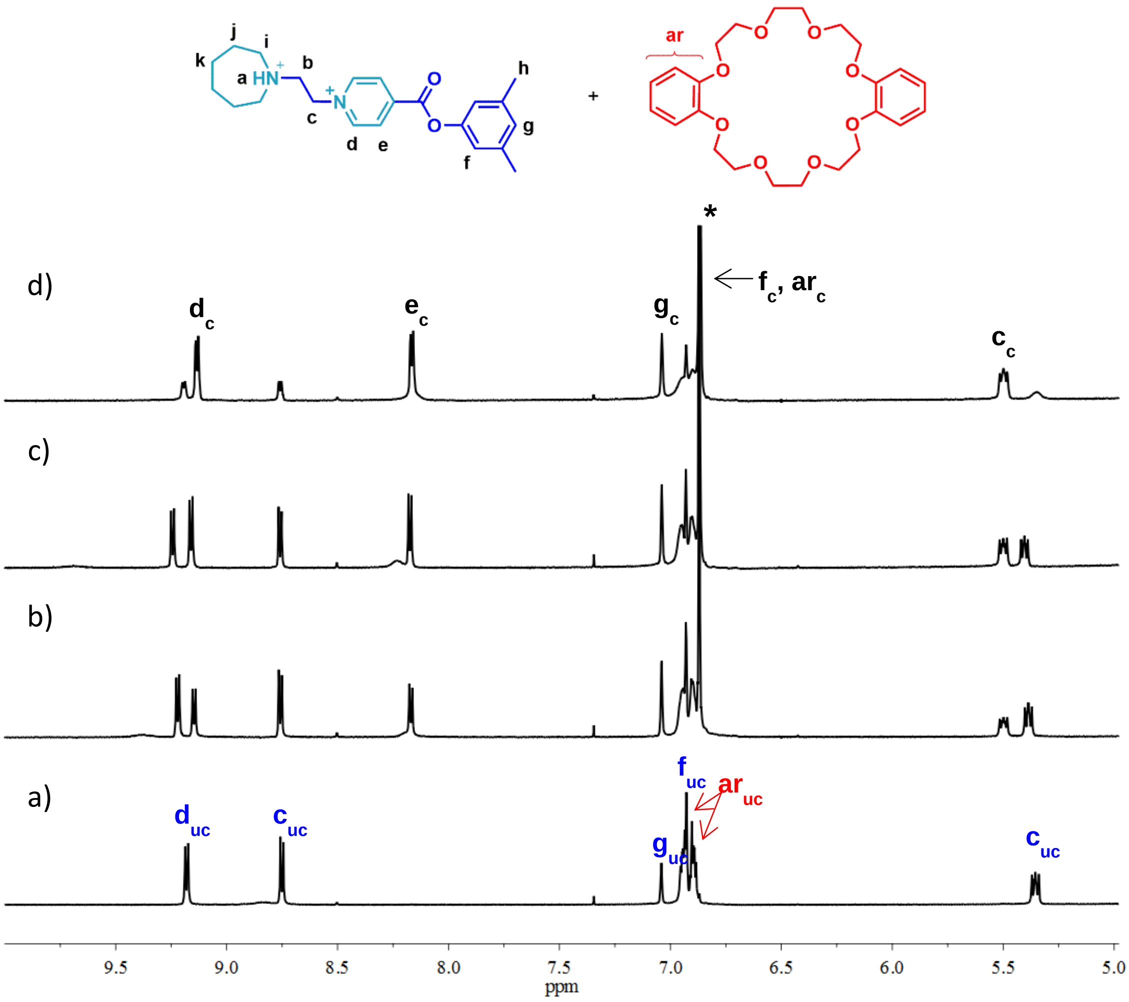

Figure S16. Partial ^{1}H NMR spectrum of an equimolar solution of guest [**1**·H]$^{2+}$ and **DB24C8** after a) 2 hours; b) 5 days; c) 10 days; d) 14 days of mixing (500 MHz, CD_3NO_2, 10 mM). (uc = free species; c = complexed species).

Association constant for complex [**1**·H⊂**DB24C8**]$^{2+}$ was determined by ^{1}H NMR employing the single point method at chemical equilibrium.

$$G + H \leftrightharpoons GH \qquad K_a = \frac{[GH]}{[G]^2}$$

$$K_a = \frac{\frac{\int [GH]}{\int [GH] + \int [G]} * [C_o]}{\left(\frac{\int [G]}{\int [GH] + \int [G]} * [C_o]\right)^2} \qquad \Delta G^o = RTlnK_a$$

Table S1. Association constant K_a and standard free energy ΔG^o for complex [**1**·H⊂**DB24C8**]$^{2+}$ in CD_3NO_2.

	C_o (M)	Integral GH	Integral G	mol χ GH	mol χ G	*K*a (M^{-1})[a]	ΔG^o (kJmol^{-1}) [b]
[**1**·H⊂**DB24C8**]$^{2+}$	0.01	3.6	1.0	0.78	0.22	1.7×10^3 ±0.2	-18.1 ±0.3

[a] The estimated error is 10%. [b] Determined at 293 K.

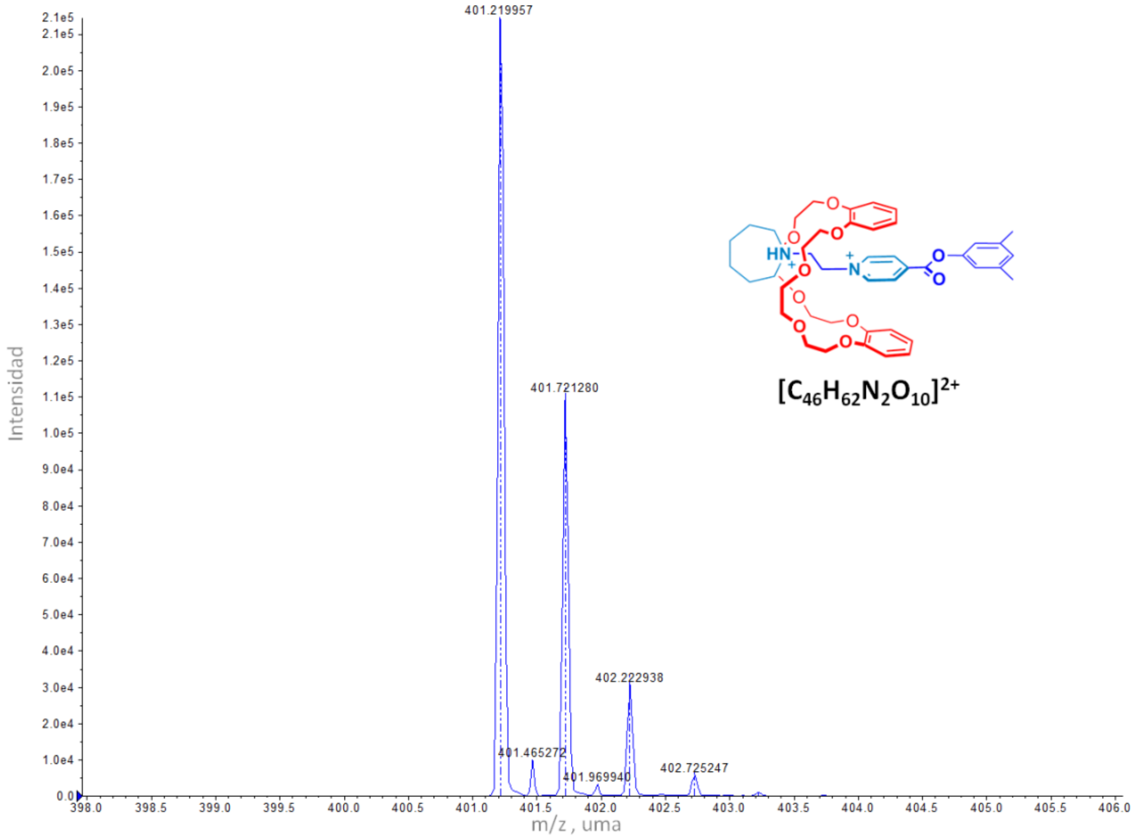

Figure S17. ESI-TOF HRMS spectrum. *m/z* found for [**1**+H+**DB24C8**]$^{2+}$ 401.2196, calculated 401.2199, error 0.7 ppm. Experimental molecular ion (continous line) and calculated isotopic profile (broken line).

Relative integration of probe H_e proton by ^1H NMR analysis led us to determine the concentration of each species through time until equilibrium was reached. With these data, association rate constant (k_{on}) was determined by a nonlinear curve fitting analysis using the next equation [S5]

$$[C]_t = \frac{[E]_0^2[C]_e e^{\left(\frac{k_{aso}t\left([E]_0^2-[C]_e^2\right)}{[C]_e}\right)} - [E]_0^2[C]_e}{[E]_0^2 e^{\left(\frac{k_{aso}t\left([E]_0^2-[C]_e^2\right)}{[C]_e}\right)} - [C]_e^2}$$

Where $[C]_t$ is the complex concentration at any time (t); $[E]_o$ initial guest concentration; $[C]_e$ complex concentration at equilibrium.

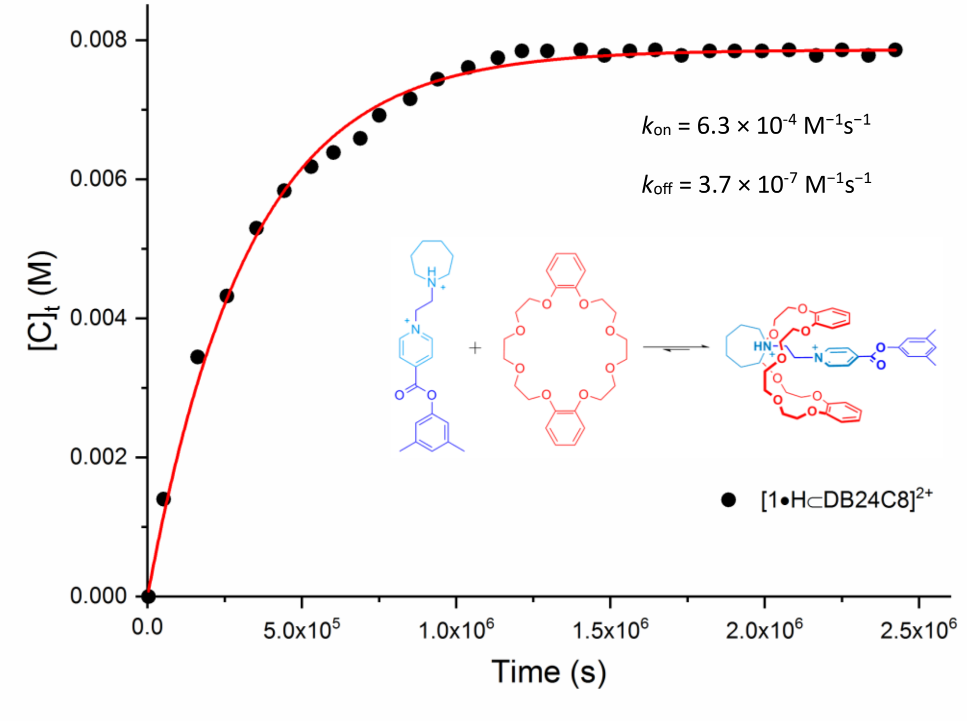

Figure S18. Experimental graphic showing the gradual formation of complex [**1**·H⊂**DB24C8**]$^{2+}$ and nonlinear curve fitting analysis.

Once the association rate constant is determined, dissociation rate constant (k_{off}) can be derived with equation

$$K_a = \frac{k_{on}}{k_{off}}$$

Table S2. Kinetic and thermodynamic parameter for complex [**1**·H⊂**DB24C8**]$^{2+}$ in CD_3NO_2 at 298 K.

	ΔG^o ($kJmol^{-1}$)	$\Delta G^{\ddagger}_{on}$ ($kJmol^{-1}$)	$\Delta G^{\ddagger}_{off}$ ($kJmol^{-1}$)
[**1**·H⊂**DB24C8**]$^{2+}$	-18.1 ± 0.3	91.2 ± 0.4	109.7 ± 0.7

Addition of one equivalent of potassium *tert*-butoxide to a nitromethane solution of compound [**1**·H⊂**DB24C8**]$^{2+}$ at chemical equilibrium, deprotonates the azepanium nitrogen rising the energy barrier high enough to transform the system into a [2]rotaxane [**1**⊂**DB24C8**]$^{+}$.

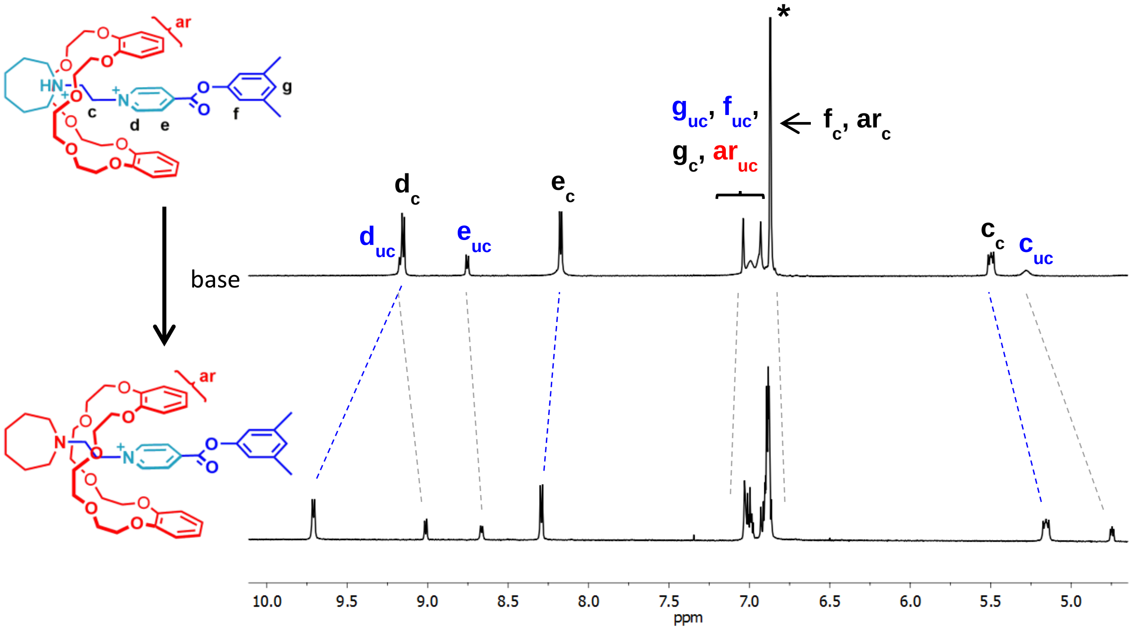

Figure S19. Partial ^1H NMR spectrum (500 MHz, CD_3NO_2, 10 mM) of an equimolar solution of guest [**1**·H]$^{2+}$ and **DB24C8** after reaching equilibrium (top) and after 1 equivalent addition of *tert*-BuOK (bottom). Lines highlight the changes on the resonances upon base addition (uc = free species; c = complexed species).

3.2 [**2**·H⊂**DB24C8**]$^{2+}$

Upon mixing two equimolar solutions of guest [**2**·H][PF_6]$_2$ and host **DB24C8** in CD_3NO_3 the ^{1}H NMR spectrum showed the formation of an interpenetrated compound in chemical equilibrium with its noncomplexed species, in clear contrast with the 14 days delay observed for guest [**1**·H][PF_6]$_2$ in the same experimental conditions (Figure S20 b). The supramolecular complex dissociates instantly after addition of one equivalent of base (Figure S20 c).

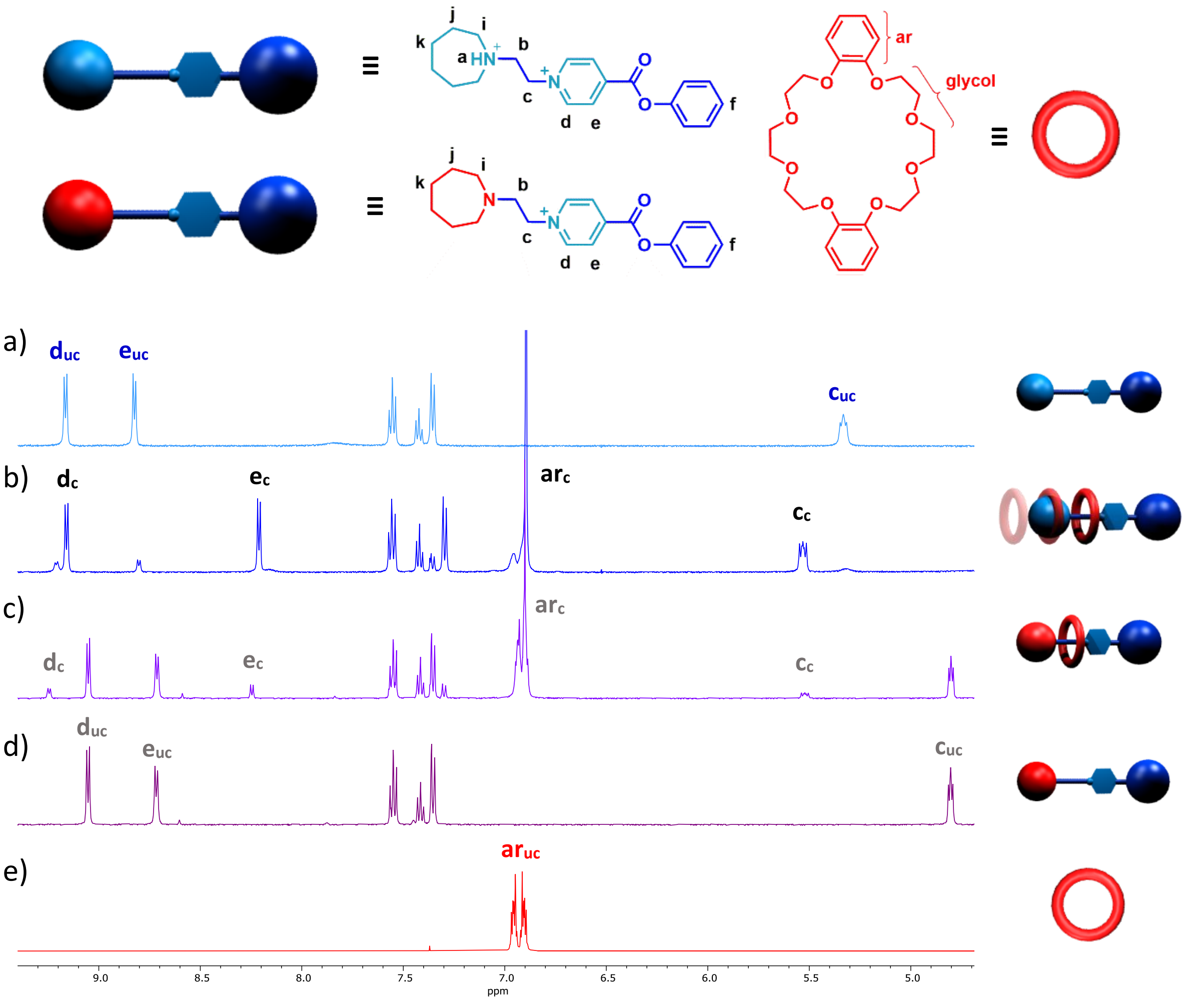

Figure S20. Partial ^{1}H NMR spectrum of: a) guest [**2**·H][PF_6]$_2$ b) equimolar mixture of [**2**·H]$^{2+}$ and **DB24C8**; c) equimolar mixture of [**2**·H]$^{2+}$ and **DB24C8** after addition of 1 equivalent of pyridine-d5; d) guest [**2**·H][PF_6]$_2$ after addition of 1 equivalent of pyridine-d5; e) **DB24C8** (500 MHz, CD_3NO_2).

Association constant for [**2**·H$\subset$**DB24C8**]$^{2+}$ was determined by ^{1}H NMR employing the single point method at chemical equilibrium.

$$G + H \leftrightharpoons GH \qquad\qquad K_a = \frac{[GH]}{[G]^2}$$

$$K_a = \frac{\frac{\int [GH]}{\int [GH] + \int [G]} * [C_o]}{\left(\frac{\int [G]}{\int [GH] + \int [G]} * [C_o]\right)^2} \qquad\qquad \Delta G^o = RTlnK_a$$

Table S3. Association constant K_a and standard free energy ΔG^o for complexes: [**2**·H$\subset$**DB24C8**]$^{2+}$ and [**2**$\subset$**DB24C8**]$^{+}$ in CD_3NO_2.

	C_o (M)	Integral GH	Integral G	mol χ GH	mol χ G	*K*a (M^{-1})[a]	ΔG^o ($kJmol^{-1}$)[b]
[**2**·H$\subset$**DB24C8**]$^{2+}$	0.011	4.1	1.0	0.80	0.20	1.9×10^3 ±0.2	-18.4 ±0.3
[**2**$\subset$**DB24C8**]$^{+}$	0.010	1.0	4.3	0.19	0.81	2.8×10^1 ±0.3	-8.1 ±0.1

[a] The estimated error is 10%. [b] Determined at 293 K.

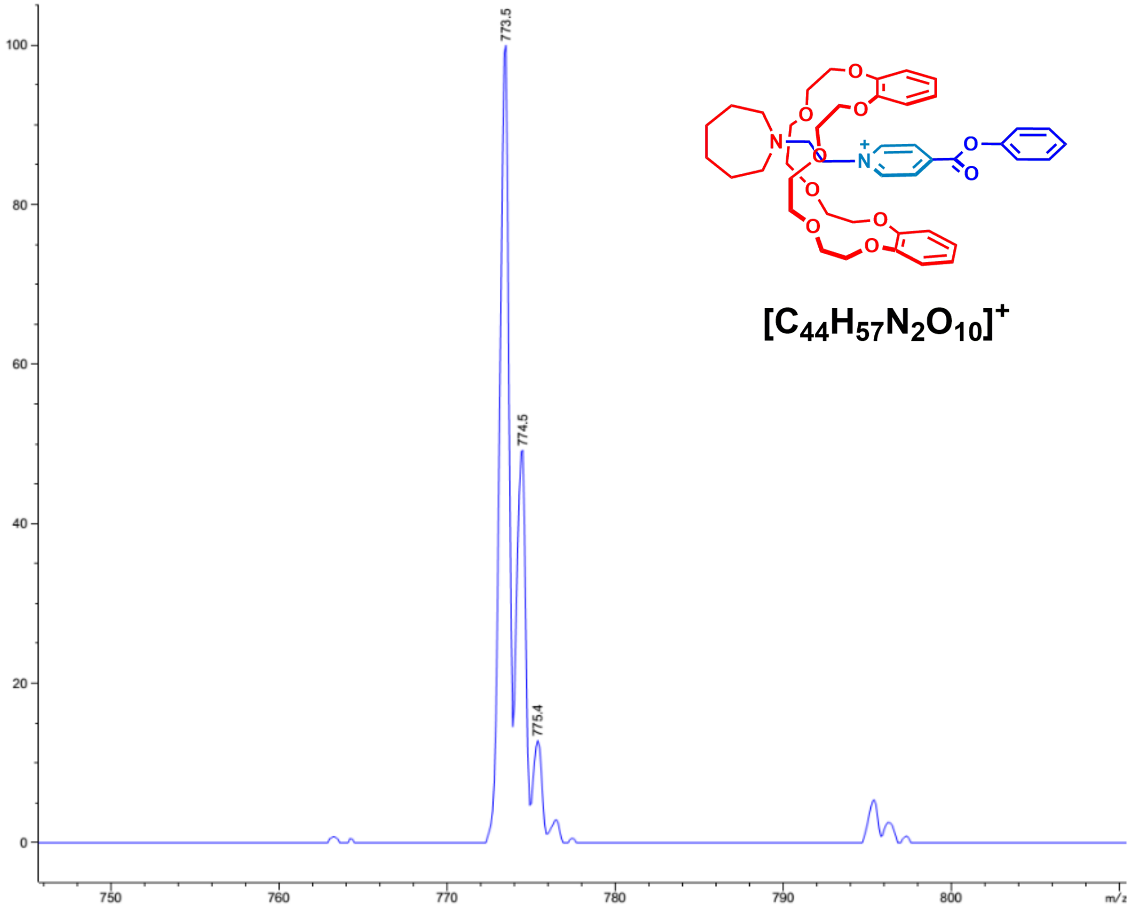

Figure S21. ESI-MS spectrum of [**2**·H⊂**DB24C8**][PF_6]$_2$. *m/z* found for [**2**+**DB24C8**]$^+$ 773.5, calculated 773.4.

3.3 [**3**·H⊂**DB24C8**]$^{2+}$

Upon mixing two equimolar solutions of guest [**3**·H][PF_6]$_2$ and host **DB24C8** in CD_3NO_3 a pale-yellow solution was observed. It took 14 days to reach equilibrium, the same period as *pseudo*-rotaxane [**3**·H⊂**DB24C8**]$^{2+}$. At this time, the ^1H NMR spectrum showed the formation of an interpenetrated compound in chemical equilibrium with its noncomplexed species (Figure S22).

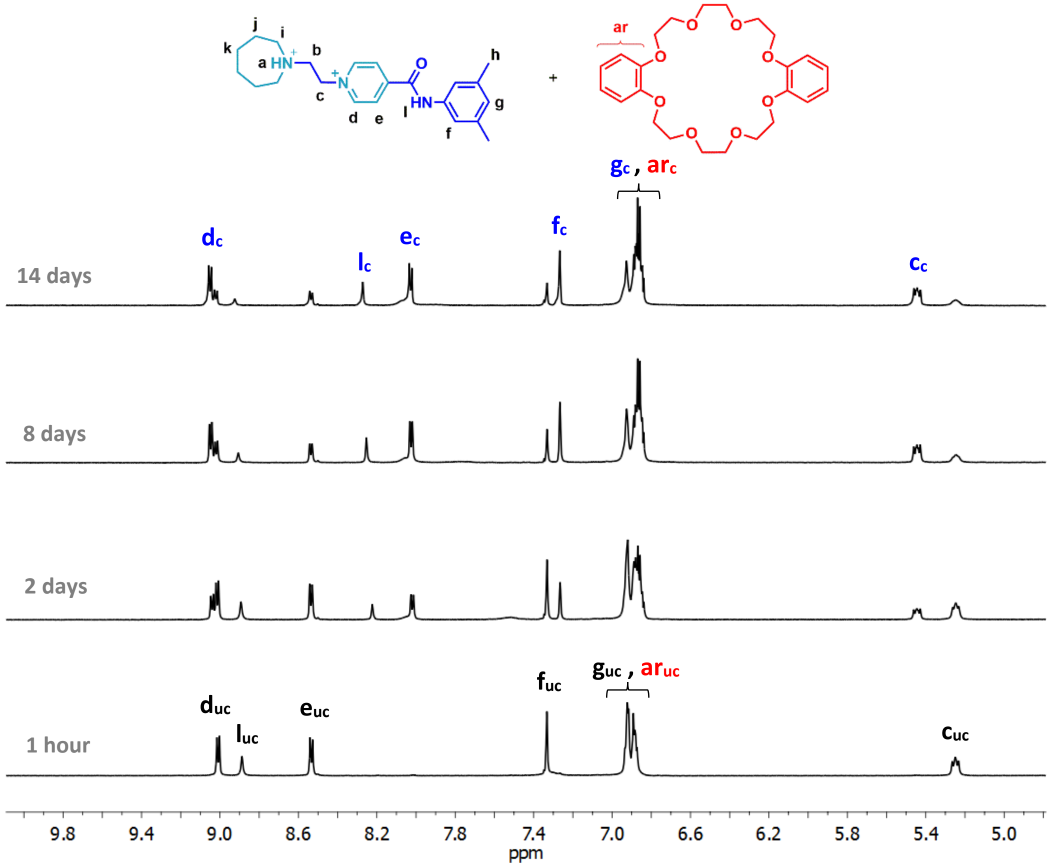

Figure S22. Partial ^1H NMR spectra (500 MHz, CD_3NO_2) showing the formation of the complex [**3**·H⊂**DB24C8**][PF_6]$_2$ from [**3**·H][PF_6]$_2$ and **DB24C8** by slippage mechanism. The equilibrium was attained approximately 14 days after mixing (uc = uncomplexed; c = complexed).

Association constant for [**3**·H⊂**DB24C8**]$^{2+}$ was determined by ^1H NMR employing the single point method at chemical equilibrium.

$$G + H \leftrightharpoons GH \qquad K_a = \frac{[GH]}{[G]^2}$$

$$K_a = \frac{\frac{\int [GH]}{\int [GH] + \int [G]} * [C_o]}{\left(\frac{\int [G]}{\int [GH] + \int [G]} * [C_o]\right)^2} \qquad \Delta G^o = RT ln K_a$$

Table S4. Association constant K_a and standard free energy ΔG^o for complex [**3**·H⊂**DB24C8**]$^{2+}$ in CD_3NO_2.

	C_o (M)	Integral GH	Integral G	mol χ GH	mol χ G	*Ka* (M^{-1})[a]	ΔG^o ($kJmol^{-1}$)[b]
[**3**·H ⊂**DB24C8**]$^{2+}$	0.005	2.64	1.0	0.72	0.28	1.9×10^3 ±0.2	-18.4 ±0.2

[a] The estimated error is 10%. [b] Determined at 293 K.

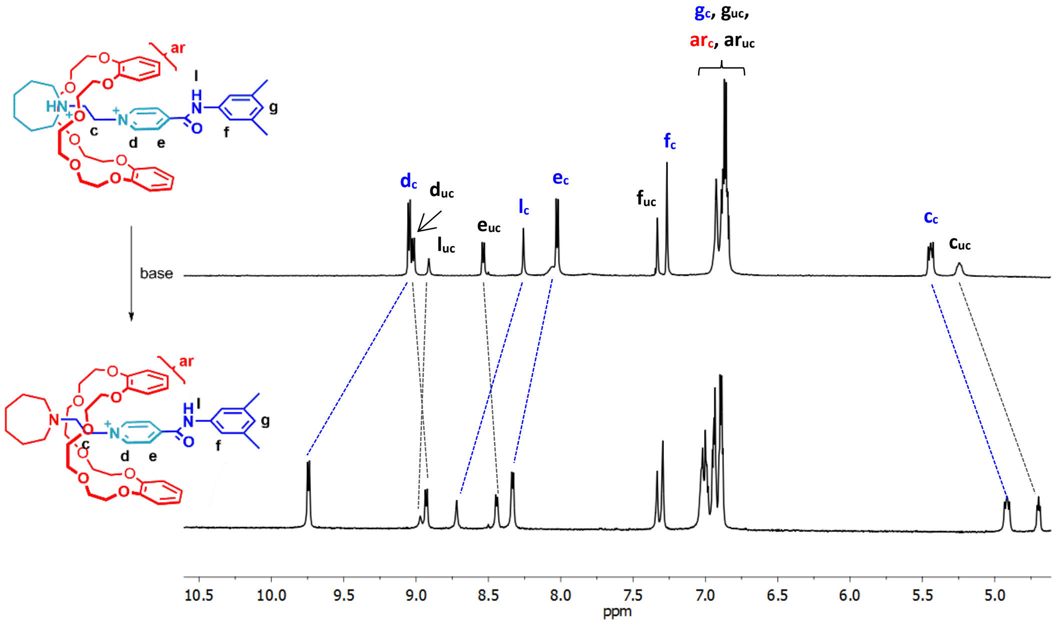

Figure S23. Partial ^{1}H NMR spectra (500 MHz, CD_3NO_2) of a solution of [**3**·H][PF_6]$_2$ and **DB24C8**: (top) when the equilibrium had been reached, (bottom) the same NMR sample after the addition of 1.2 equivalents of *tert*-BuOK; showing that the deprotonated complex [**3**⊂**DB24C8**][PF_6] does not dissociate in their individual components. The system was monitored for one month. Lines highlight the changes on the resonances upon base addition (uc = uncomplexed; c = complexed).

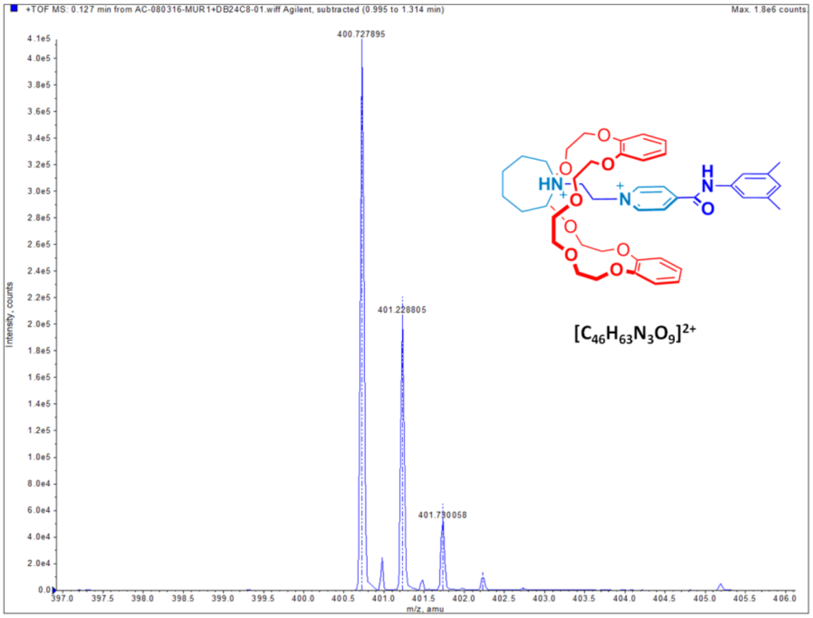

Figure S24. ESI-HRMS spectrum of [**3**·H⊂**DB24C8**][PF_6]$_2$. *m/z* found for [**3**+H+**DB24C8**]$^{2+}$ 400.7279, calculated 400.7277, error 0.6 ppm. Experimental molecular ion (continous line) and calculated isotopic profile (broken line).

4. Computational analysis.

All calculations were performed using Gaussian 09, Revision D.01 [S6] employing the range-separated hybrid exchange-correlation density functional approximation with damped atom-atom dispersion, ωB97XD, and the orbital basis set TZVP [S7]. The SCF tolerance was set to 10^{-6}, and DIIS was used to expedite SCF convergence. The full geometry optimization of all reported structures was carried out using Berny's method in internal redundant coordinates with BFGS Hessian update, and without any geometrical constraints. Implicit solvation using nitromethane was implemented through the SMD solvation model [S8]. Frequency analysis was conducted following the same methodological procedure as the optimization. The presence of only real frequencies in the results confirms that the structures correspond to a minimum on the potential energy surface.

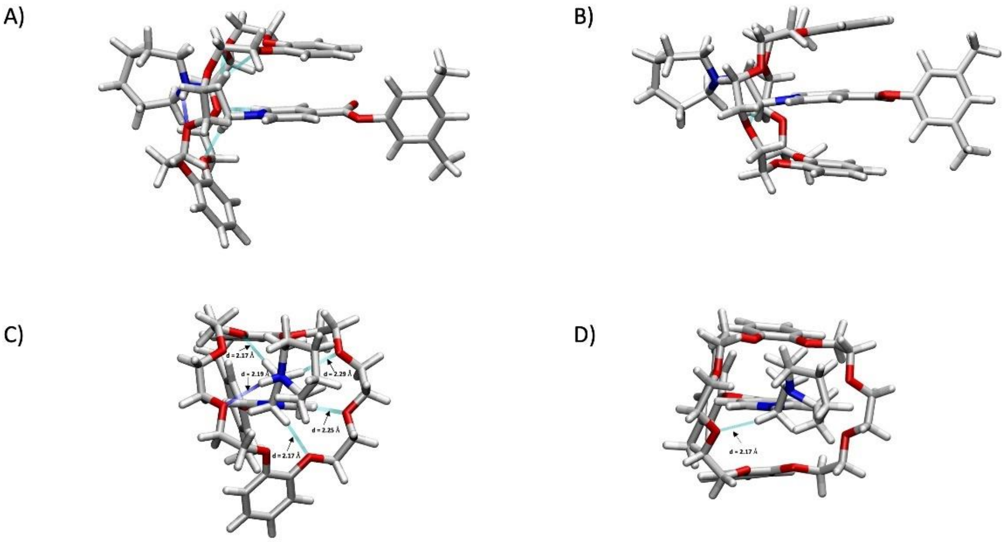

Figure S25. In A) and B), the $[\mathbf{1}\cdot\mathrm{H}\subset\mathbf{DB24C8}]^{2+}$ (pseudo-rotaxane) and $[\mathbf{1}\subset\mathbf{DB24C8}]^{+}$ (rotaxane) structures are depicted in licorice representation, revealing the formation of two types of hydrogen bonds: C-H···O (turquoise line) and N-H···O (blue line), where the bond distances (d) are illustrated in the frontal views for each complex in C) and D) respectively

Table S5. Cartesian coordinates for the minimum energy structure of *pseudo*-rotaxane [**1**·H⊂**DB24C8**]$^{2+}$.

Pseudo-rotaxane[**1·H⊂ DB24C8**]$^{2+}$											
O	-0.03077	-3.26933	6.33741	C	0.71726	-1.81729	8.11443	H	-2.32852	-6.04610	7.20151
O	-0.92628	-5.73795	5.14838	C	-1.12007	-1.10565	9.47997	H	-2.46408	-6.28973	8.95314
O	-3.57316	-6.84061	5.44830	C	-1.99374	-1.43367	10.53594	H	-0.59942	-4.34311	9.87850
O	-4.51350	-7.74074	7.66984	C	-2.73430	-6.68202	12.26262	H	-0.45472	-6.03874	10.35205
O	-3.03960	-7.93614	10.24309	C	-2.61247	-3.02162	12.20121	H	-0.17731	-5.05522	7.59609
O	-0.24027	-2.08089	9.13339	C	-2.26691	-4.42147	12.61955	H	-1.28797	-7.72328	4.88877
O	-1.84482	-2.67922	11.05403	C	-13.12451	-1.68743	7.34309	H	-0.99671	-6.83747	3.38894
O	-2.87842	-5.36486	11.76069	C	-12.47207	-5.48762	4.10378	H	-3.19739	-5.60755	3.81672
O	-8.69265	-3.70117	6.64475	C	-11.91534	-4.53210	5.12139	H	-3.33770	-7.34506	3.45349
O	-8.97214	-4.87501	8.54580	C	-12.72343	-3.59709	5.75521	H	-3.68268	-2.95091	11.98465
N	-4.05812	-4.25377	8.13189	C	-12.21810	-2.69775	6.69693	H	-2.37382	-2.33581	13.01940
N	-0.57312	-5.70482	8.29059	C	-10.86571	-2.74860	7.00991	H	-2.63555	-4.56269	13.64283
C	1.83226	-7.58940	9.44641	C	-10.55760	-4.57169	5.44408	H	-1.17818	-4.55688	12.62929
C	2.23257	-6.28202	10.13724	C	-10.06870	-3.68677	6.37809	H	-3.01521	-9.38270	8.83290
C	-7.17014	-6.53089	4.73935	C	-8.26744	-4.33382	7.74067	H	-5.52564	-9.35636	8.47435
C	-7.67435	-7.03031	5.92317	C	-0.16318	-7.10281	7.91503	H	-5.58785	-7.86817	9.44191
C	-6.80783	-7.45641	6.92916	C	-0.10037	-5.27952	9.65425	H	0.86000	-1.43559	6.02917
C	-5.79178	-6.45819	4.54186	C	1.31698	-7.42370	8.01635	H	-1.80239	-3.20604	5.27385
C	-2.14177	1.07654	9.35047	H	1.42696	-8.37372	7.48894	H	-0.39065	-2.53159	4.43985
C	-2.99953	0.75009	10.38098	H	1.90708	-6.69350	7.45579	H	-0.79805	-1.37744	6.64823
C	-2.92458	-0.50770	10.97652	H	2.69001	-8.26469	9.44425	H	-4.02581	-9.75074	10.24097
C	-1.20167	0.15059	8.89969	H	1.05366	-8.09220	10.02818	H	-3.04070	-6.70625	13.31577
C	-6.18349	-4.71502	9.03670	H	3.28102	-6.05881	9.92423	H	-3.68255	-8.56828	12.09445
C	-4.81750	-4.68269	9.15709	H	2.15876	-6.41648	11.21930	H	-0.98698	-4.57812	3.42785
C	-6.77130	-4.28104	7.85491	H	1.74776	-4.70434	8.74732	H	1.17219	-0.83499	8.26121
C	-5.96990	-3.84010	6.81431	H	1.58748	-4.25215	10.42055	H	-1.68523	-7.00000	12.20776
C	-4.60389	-3.84785	6.97761	H	-7.83853	-6.19402	3.95656	H	-4.61334	-7.22479	11.40425
C	-2.59445	-4.22348	8.30292	H	-5.40367	-6.07190	3.60909	H	1.49668	-2.56529	8.25011
C	-2.06054	-5.64120	8.17484	H	-8.74355	-7.09255	6.08345	H	0.60514	-4.75036	4.19025
C	1.40566	-5.06801	9.71929	H	-7.21575	-7.83890	7.85457	H	-13.48599	-0.96620	6.60590
C	0.15482	-1.91303	6.71963	H	-2.19004	2.05129	8.88142	H	-13.99995	-2.17147	7.78084
C	-0.72676	-3.33856	5.10076	H	-0.53820	0.42991	8.09349	H	-12.60836	-1.13693	8.13017
C	-0.46619	-4.63423	4.38947	H	-3.73240	1.46389	10.73567	H	-11.98498	-5.35024	3.13546
C	-1.48146	-6.78304	4.36818	H	-3.59535	-0.75433	11.78758	H	-12.30430	-6.52342	4.40743
C	-2.96934	-6.61461	4.18099	H	-6.78165	-5.06670	9.86498	H	-13.54370	-5.33969	3.96868
C	-4.92286	-6.87594	5.53629	H	-4.28975	-4.97965	10.05766	H	-13.78002	-3.56009	5.51036
C	-5.43708	-7.37549	6.75032	H	-6.38384	-3.49918	5.87784	H	-10.43384	-2.06865	7.73464
C	-4.94970	-8.48415	8.80037	H	-3.91906	-3.52705	6.20669	H	-9.89130	-5.28223	4.97012
C	-3.72447	-8.95876	9.54561	H	-2.40540	-3.79173	9.28096	H	-0.73950	-7.76079	8.56676
C	-3.60533	-7.64505	11.50681	H	-2.18399	-3.57042	7.53706	H	-0.50550	-7.23089	6.89331

Table S6. Cartesian coordinates for the minimum energy structure of rotaxane [**1**⊂**DB24C8**]$^{+}$.

Rotaxane[**1**⊂ **DB24C8**]$^{+}$											
O	-0.73043	-2.55796	6.44293	C	-1.25366	-0.82145	8.00593	H	-1.56822	-6.34037	7.04084
O	-1.70894	-4.83670	4.98216	C	-3.43730	-1.30953	8.89882	H	-1.91695	-6.95178	8.65537
O	-3.72068	-6.90108	5.72816	C	-4.13758	-1.94977	9.94503	H	-0.30800	-5.81097	10.30852
O	-4.24761	-8.08788	8.00214	C	-2.54864	-6.58029	12.51395	H	0.20741	-7.46512	10.07576
O	-2.65079	-7.94775	10.56067	C	-3.98115	-3.24964	11.93918	H	-1.33741	-6.87941	4.85255
O	-2.12155	-1.61724	8.80545	C	-3.06519	-4.26904	12.56376	H	-1.62883	-6.04519	3.31364
O	-3.40971	-2.79522	10.71721	C	-11.09527	0.20265	6.49138	H	-3.95384	-5.49654	4.21065
O	-3.27326	-5.51602	11.93472	C	-12.28692	-4.46702	5.02135	H	-3.64543	-7.16602	3.68187
O	-7.74567	-3.36963	6.52748	C	-11.28259	-3.48848	5.56317	H	-4.95301	-3.72051	11.76837
O	-8.24984	-4.21886	8.54990	C	-11.62487	-2.16028	5.79705	H	-4.11531	-2.40069	12.61608
N	-3.32001	-4.66637	8.19449	C	-10.70158	-1.23248	6.27841	H	-3.30951	-4.34558	13.63058
N	0.07293	-6.27386	8.32154	C	-9.40255	-1.65650	6.53769	H	-2.01855	-3.95265	12.47546
C	2.69972	-8.06302	8.83166	C	-9.97629	-3.89935	5.82501	H	-2.43137	-9.37296	9.13372
C	2.90054	-7.05680	9.96823	C	-9.07130	-2.97792	6.30640	H	-4.98135	-9.76214	8.97063
C	-7.40075	-6.93649	5.57530	C	-7.45325	-3.96428	7.68943	H	-5.14545	-8.23760	9.87080
C	-7.67845	-7.54178	6.78266	C	0.63482	-7.33073	7.48572	H	-0.47230	-0.53576	6.06938
C	-6.63424	-7.95310	7.60950	C	0.38973	-6.43011	9.74587	H	-1.02205	-2.45398	4.39656
C	-6.07775	-6.73008	5.18764	C	2.15366	-7.47021	7.53118	H	0.67227	-2.52967	4.92636
C	-5.48407	-0.22611	8.21737	H	2.42528	-8.13191	6.70430	H	-2.14638	-1.11580	6.04434
C	-6.16935	-0.85638	9.23662	H	2.62017	-6.50187	7.32263	H	-3.29336	-9.91352	10.57932
C	-5.49338	-1.71068	10.10532	H	3.65550	-8.55609	8.63638	H	-2.72124	-6.61141	13.59749
C	-4.11763	-0.44640	8.05393	H	2.01494	-8.85272	9.15725	H	-2.57979	-8.70850	12.48032
C	-5.54602	-4.98624	8.89422	H	3.86259	-6.55242	9.83827	H	0.09870	-4.64159	3.99196
C	-4.19852	-5.19027	9.06224	H	2.97500	-7.61170	10.90808	H	-1.52642	0.23520	8.07522
C	-5.98822	-4.24802	7.80691	H	2.04528	-5.13364	9.45065	H	-1.47275	-6.45356	12.34072
C	-5.06642	-3.76190	6.89302	H	1.81648	-5.60449	11.11891	H	-4.11824	-7.94421	12.02952
C	-3.72671	-3.98082	7.11749	H	-8.20014	-6.61937	4.91745	H	-0.26979	-0.94832	8.45811
C	-1.87655	-4.80365	8.47327	H	-5.88091	-6.25686	4.23652	H	0.21435	-4.89167	5.74850
C	-1.36641	-6.18731	8.09842	H	-8.70200	-7.70647	7.09572	H	-11.09112	0.74594	5.54254
C	1.80959	-5.98537	10.09422	H	-6.86411	-8.43119	8.55101	H	-12.10132	0.27814	6.90731
C	-1.18000	-1.22318	6.55073	H	-5.99819	0.44462	7.54013	H	-10.40288	0.70672	7.16653
C	-0.33883	-2.90405	5.12670	H	-3.59478	0.05593	7.25231	H	-12.10743	-4.65390	3.95924
C	-0.36425	-4.40003	4.95626	H	-7.23083	-0.69131	9.37343	H	-12.21972	-5.42630	5.53756
C	-1.92568	-6.08990	4.36897	H	-6.04037	-2.18979	10.90487	H	-13.30334	-4.08654	5.12704
C	-3.39505	-6.41017	4.43232	H	-6.23964	-5.38400	9.62031	H	-12.64208	-1.83656	5.60160
C	-5.02981	-7.11184	6.01066	H	-3.78235	-5.71663	9.91031	H	-8.65513	-0.97080	6.91910
C	-5.31392	-7.74437	7.24126	H	-5.36665	-3.19134	6.02738	H	-9.67139	-4.92671	5.66397
C	-4.49227	-8.81087	9.20470	H	-2.95005	-3.62258	6.45896	H	0.19024	-8.30893	7.74726
C	-3.16816	-9.07720	9.88344	H	-1.75818	-4.58544	9.53243	H	0.33901	-7.11546	6.45647
C	-3.02845	-7.87766	11.92128	H	-1.36487	-4.03751	7.89849				

5. Transesterification reaction.

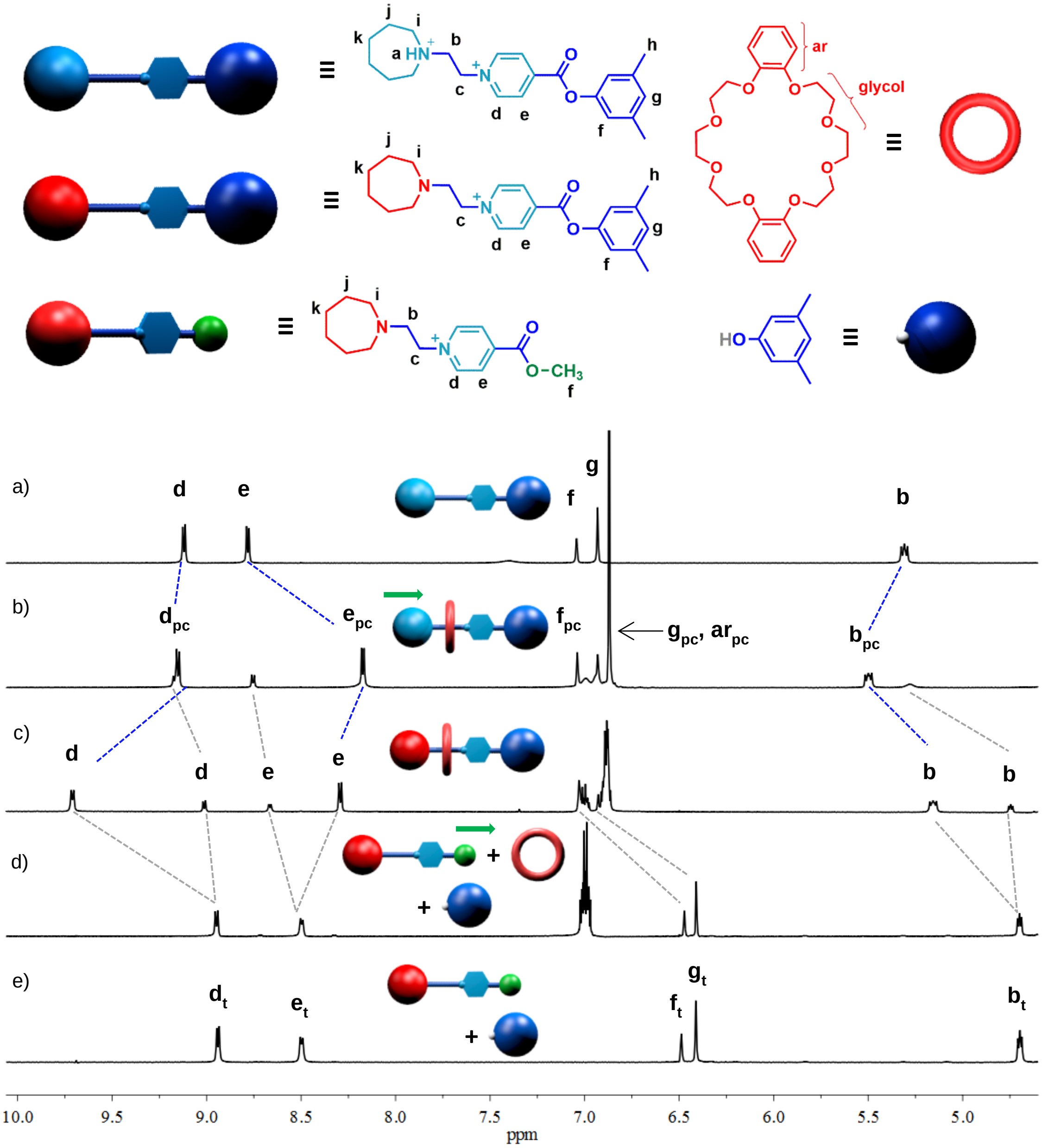

Figure S26. Partial ^{1}H NMR spectra (500 MHz, CD_3NO_2) of a) [**1**·H][PF_6]$_2$; b) an equimolar mixture of [**1**·H][PF_6]$_2$ and **DB24C8** at equilibrium; c) the previous system after addition of one equivalent of base; d) the previous system after addition of 100 equivalents of methanol; e) a [**1**·H][PF_6]$_2$ solution after addition of one equivalent of base and 100 equivalents of methanol. (fp = free protonated; pc = protonated complex; dc = deprotonated complex; fd = free deprotonated; t=transesterification products).

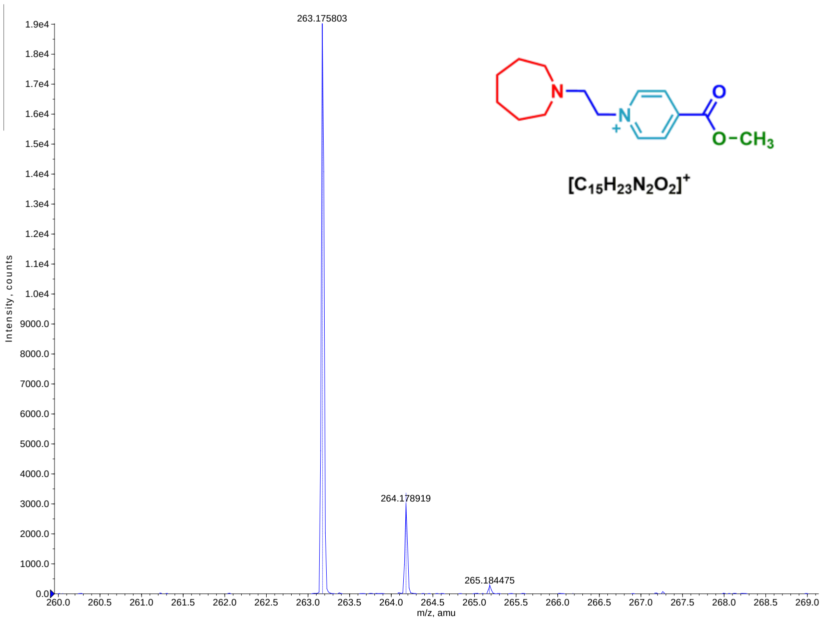

Figure S27. ESI-TOF HRMS spectrum of methyl-ester compound. *m/z* found for $[C_{15}H_{23}N_2O_2]^+$ 263.1758, calculated 263.1754, error 1.5 ppm. Experimental molecular ion (continous line) and calculated isotopic profile (broken line).

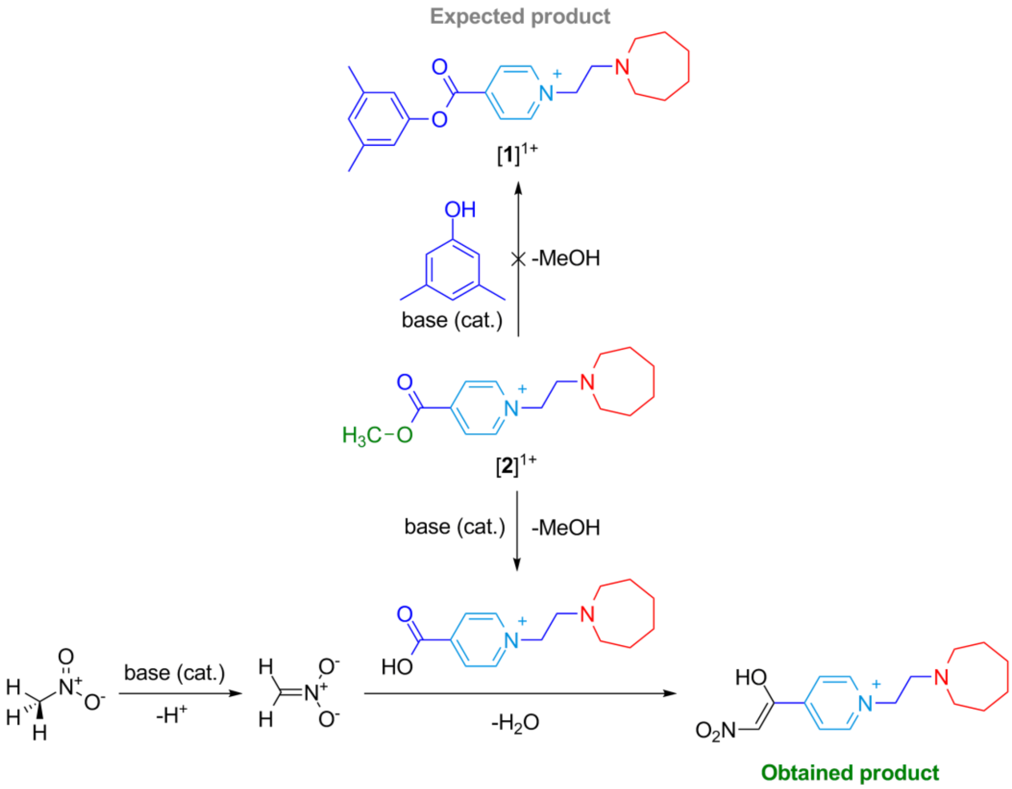

Figure S28. In the attempt to regenerate compound [**1**·H]$^+$ by the addition of 3,5-dimethyl phenol and base, the methyl-ester compound suffers hydrolysis and react with the nitroalkane derived from nitromethane by a Henry nitro-aldolic mechanism to form an unexpected, nitrated product.

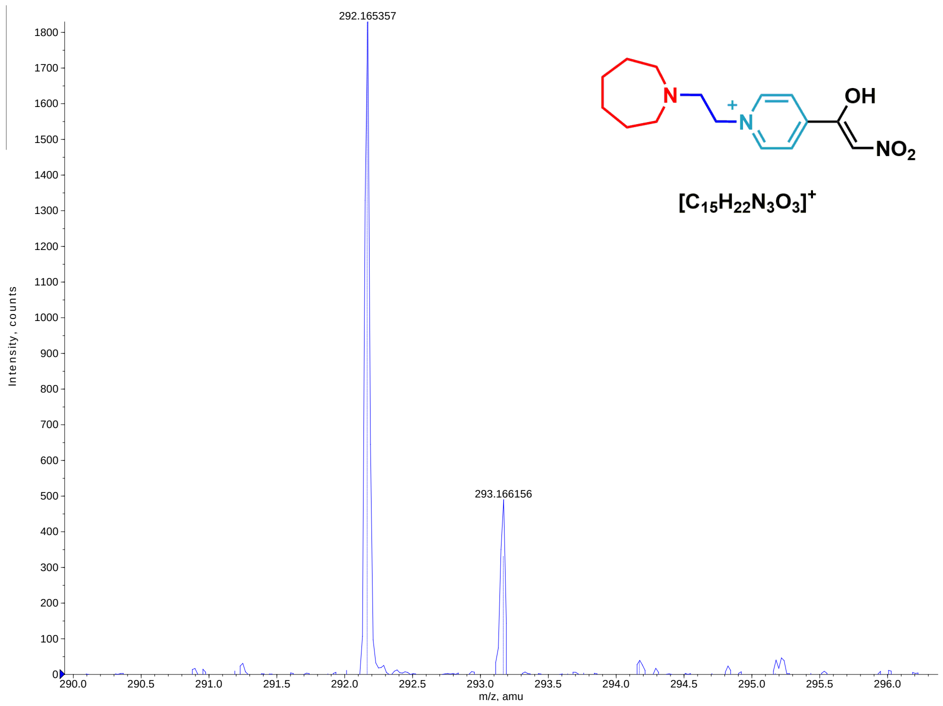

Figure S29. ESI-TOF HRMS spectrum of the unexpected, nitrated product. *m/z* found for [nitrocompound]$^+$ 292.1654, calculated 292.1656, error 0.7 ppm. Experimental molecular ion (continous line) and calculated isotopic profile (broken line).

6. References.

[S1] C. Chiappe, C. S. Pomelli, S. Rajamani, *J. Phys. Chem. B*, **2011**, *115*, 9653-9661.

[S2] R. Lakhan, O. P. Singh, *Arch. Pharm.*, **1985**, *318*, 228-238; L. H. Amundsen, K. W. Krantz, *J. Am. Chem. Soc.*, **1941**, *63*, 305-307; F. Cortese, *J. Am. Chem. Soc.*, **1936**, *58*, 191-192.

[S3] L. M. Jackman, M. M. Petrei, B. D. Smith, *J. Am. Chem. Soc.*, **1991**, *113*, 3451-3458.

[S4] Wasa, M.; Worrell, B. T.; Yu, J.-Q., *Angew. Chem. Int. Ed.,* **2010**, *49*, 1275-1277.

[S5] K. J. Laidler, *Chemical Kinetics*, 2nd Edition, McGraw-Hill, **1965**, 19-21.

[S6] Gaussian 09, Revision D.01, M. J. Frisch, G. W. Trucks, H. B. Schlegel, G. E. Scuseria, M. A. Robb, J. R. Cheeseman, G. Scalmani, V. Barone, B. Mennucci, G. A. Petersson, H. Nakatsuji, M. Caricato, X. Li, H. P. Hratchian, A. F. Izmaylov, J. Bloino, G. Zheng, J. L. Sonnenberg, M. Hada, M. Ehara, K. Toyota, R. Fukuda, J. Hasegawa, M. Ishida, T. Nakajima, Y. Honda, O. Kitao, H. Nakai, T. Vreven, J. A. Montgomery, Jr., J. E. Peralta, F. Ogliaro, M. Bearpark, J. J. Heyd, E.

Brothers, K. N. Kudin, V. N. Staroverov, T. Keith, R. Kobayashi, J. Normand, K. Raghavachari, A. Rendell, J. C. Burant, S. S. Iyengar, J. Tomasi, M. Cossi, N. Rega, J. M. Millam, M. Klene, J. E. Knox, J. B. Cross, V. Bakken, C. Adamo, J. Jaramillo, R. Gomperts, R. E. Stratmann, O. Yazyev, A. J. Austin, R. Cammi, C. Pomelli, J. W. Ochterski, R. L. Martin, K. Morokuma, V. G. Zakrzewski, G. A. Voth, P. Salvador, J. J. Dannenberg, S. Dapprich, A. D. Daniels, O. Farkas, J. B. Foresman, J. V. Ortiz, J. Cioslowski, and D. J. Fox, Gaussian, Inc., Wallingford CT, **2013**.

[S7] J.-D. Chai and M. Head-Gordon, *Phys. Chem. Chem. Phys.*, **2008**, *10*, 6615-6620.

[S8] A. V. Marenich, C. J. Cramer, D. G. Truhlar, *J. Phys. Chem. B*, **2009**, *113,* 6378-96.
